# Synthesis of chiral Cu(II) complexes from pro-chiral Schiff base ligand and investigation of their catalytic activity in the asymmetric synthesis of 1,2,3-triazoles

**DOI:** 10.1038/s41598-024-60930-w

**Published:** 2024-05-08

**Authors:** Fatemeh Ajormal, Rahman Bikas, Nader Noshiranzadeh, Marzieh Emami, Anna Kozakiewicz-Piekarz

**Affiliations:** 1 Department of Chemistry, Faculty of Science, University of Zanjan, Zanjan, 45371-38791 Iran; 2 Department of Chemistry, Faculty of Science, Imam Khomeini International University, Qazvin, 34148-96818 Iran; 3 Department of Biomedical and Polymer Chemistry, Faculty of Chemistry, Nicolaus Copernicus University in Torun, 87-100, Torun, Poland

**Keywords:** Chemistry, Catalysis, Coordination chemistry, Inorganic chemistry, Organic chemistry, Chemical synthesis

## Abstract

A pro-chiral Schiff base ligand (HL) was synthesized by the reaction of 2-amino-2-ethyl-1,3-propanediol and pyridine-2-carbaldehyde in methanol. The reaction of HL with CuCl_2_·2H_2_O and CuBr_2_ in methanol gave neutral mononuclear Cu(II) complexes with general formula of [Cu(HL)Cl_2_] (**1**) and [Cu(HL)Br_2_] (**2**), respectively. By slow evaporation of the methanolic solutions of **1** and **2**, their enantiomers were isolated in crystalline format. The formation of pure chiral crystals in the racemic mixture was amply authenticated by single crystal X-ray analysis, which indicated that S-[Cu(HL)Cl_2_], R-[Cu(HL)Cl_2_], and S-[Cu(HL)Br_2_] are crystallized in chiral *P2*_*1*_*2*_*1*_*2*_*1*_ space group of orthorhombic system. Preferential crystallization was used to isolate the R and S enantiomers as single crystals and the isolated compounds were also studied by CD analysis. Structural studies indicated that the origin of the chirality in these compounds is related to the coordination mode of the employed pro-chiral ligand (HL) because one of its carbon atoms has been converted to a chiral center in the synthesized complexes. Subsequently, these complexes were used in click synthesis of a β-hydroxy-1,2,3-triazole and the results of catalytic studies indicated that **1** and **2** can act as enantioselective catalysts for the asymmetric synthesis of β-hydroxy-1,2,3-triazole product under mild condition. This study illustrates the significant capacity of the use of pro-chiral ligands in preparing chiral catalysts based on complexes which can also be considered as an effective approach to cheap chiral catalysts from achiral reagents.

## Introduction

The research on chirality and controlling pure stereochemistry during the synthesis of chiral molecules is of great importance in chemistry, pharmacy, and materials science^1–3^. The use of asymmetric synthesis in the presence of chiral catalytic systems is one of the effective methods for stereoselective and enantioselective synthesis^4–10^. Chiral complexes are the most important materials that have been used as catalysts for asymmetric synthesis^11–14^. Due to this, the design and synthesis of chiral complexes is an important research area in the development of catalysts for asymmetric synthesis. The reaction of metal salt precursors with carefully tailored chiral ligands is the most popular method for the synthesis of chiral complexes^15–20^. Generating metal-centered helical chirality through the self assembly of metal salts with suitable ligands is also a powerful, but not so controllable, method for the synthesis of chiral coordination polymers^21,22^. Since most chiral precursors for the synthesis of chiral ligands are expensive, the synthesis of chiral complexes by using inexpensive pro-chiral ligand and introducing chiral center during the synthesis of the complex is one of the attractive and also challenging ways for cost-effective synthesis of chiral catalysts^23–25^. The formation and synthesis of conglomerates from pro-chiral ligands and the isolation of chiral products via preferential crystallization process are relatively rare phenomena. Nevertheless, several spontaneous resolutions of complexes, including the first chiral complex [Co(en)_2_(ox)]Br_3_ by Werner^26^, and several of those have been reported in recent years^27–31^.

The extensive biological activities of 1,2,3-triazoles (such as antibacterial characteristics, anti-inflammatory, and anticancer properties) have introduced them as an important category of synthons and building blocks in medicinal chemistry and pharmaceutical science^32–36^. The biological evaluation demonstrated that introducing chiral triazole cores in the scaffold of biological materials may allow greater results in enantioselective bioactivity^37,38^. Synthetic organic chemists usually consider the direct use of efficient chiral catalysts^39–42^, chiral raw materials^43^, or chiral environment^44–46^ for enantioselective synthesis of triazole derivatives. Thus, the process for the synthesis of chiral triazoles remains partly expensive as optically pure starting materials are required in these processes. Despite the high importance and wide applications of 1,2,3-triazoles, there are limited reports about their asymmetric synthesis from pro-chiral materials^47–49^. Especially, β-hydroxy-1,2,3-triazoles, obtained by the reaction of epoxide-azide-alkyne cycloaddition reaction, have usually been produced from a racemic mixture of epoxides^50–52^ and to the best of our knowledge, there is not any report about their enantioselective synthesis. Recently, enantioselective separation of β-hydroxy-1,2,3-triazoles by HPLC has been reported by Alvarenga et al.^53^.

**Figure 1:**
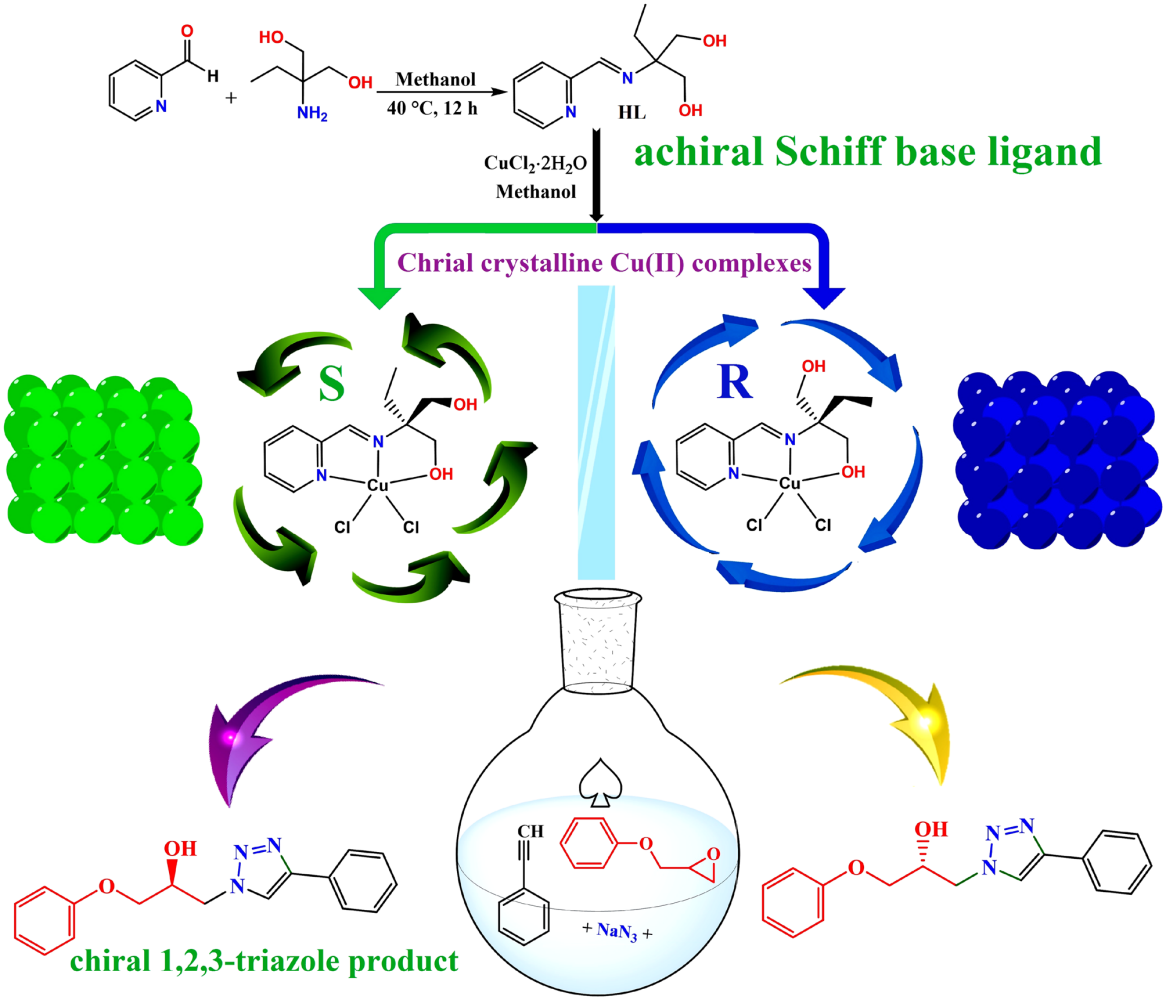
Synthesis procedure and structures of the ligand, Cu(II) complexes and triazole product.

From an availability and economic standpoint, a multi-step production process to synthesize the chiral ligands is the common limitation in the design and synthesis of chiral transition metal catalysts. This process is often time-consuming and expensive pragmatic. Moreover, the utilization of precious metal salts in the synthesis of these catalysts significantly increases their synthesis cost. Lowering the cost of chiral technology is also an old and permanent objective for chemists in this regard^54,55^. The synthesis and separation of chiral products from achiral materials is a very attractive and meanwhile unique phenomenon. In this report, we describe a straightforward design and synthesis of chiral Cu(II) complexes from an achiral Schiff base ligand that can be synthesized in a single step from readily available and inexpensive starting compounds. The employed ligand, HL, contains two alcoholic arms and only one of them is coordinated to the metal ion, consequently, the carbon attached to the alcohol arms is postulated as a potential site for asymmetric center creating and chirality induction (see Fig. 1). The S- and R-enantiomers of the synthesized Cu(II) complexes have been isolated as pure chiral enantiomers during the formation of single crystals. Since copper-based catalysts have considerable benefits such as diverse functions, affordable, easy handling, inexpensive, and non-toxicity, and also in continuing to our previous reports on the synthesis of β-hydroxy-1,2,3-triazole compounds in the presence of Cu(II) catalysts, we interested to investigate the catalytic activity of these compounds in the asymmetric synthesis of triazoles. The introduced ligand and technique in this report can open new insights for the design and synthesis of chiral compounds from pro-chiral ligands.

## Experimental

### Materials and instrumentation

The details of materials and instrumentations together with X-ray analysis are presented in the supporting file. The crystallographic data of the synthesized compounds are summarized in Table 1.

**Table 1:** Important crystallographic data for the compounds **1**, **2**, and **T1.**

Compound	S-[Cu(HL)Cl_2_] (**1**)	R-[Cu(HL)Cl_2_] (**1**)	S-[Cu(HL)Br_2_] (**2**)	**T1**
CCDC number	2288813	2288814	2288815	1921722
Net formula	C_11_H_16_Cl_2_CuN_2_O_2_	C_11_H_16_Cl_2_CuN_2_O_2_	C_11_H_16_Br_2_CuN_2_O_2_	C_17_H_17_N_3_O_2_
*M*_r_/g mol^−1^	342.70	342.70	431.62	295.33
Crystalsize/mm	0.61 × 0.29 × 0.15	0.37 × 0.35 × 0.29	0.20 × 0.20 × 0.14	0.51 × 0.12 × 0.04
*T*/K	293	293	100	293
Radiation	Mo *K*α	Mo *K*α	Cu *K*α	Mo *K*α
Crystalsystem	Orthorhombic	Orthorhombic	Orthorhombic	Monoclinic
Crystalshape, color	Plate, green	Block, green	Block, green	Needle, colorless
space group	*P*2_1_2_1_2_1_	*P*2_1_2_1_2_1_	*P*2_1_2_1_2_1_	*P*2_1_
*a*/Å	9.6111(3)	9.6661(13)	9.7775(7)	5.6181(12)
*b*/Å	11.6447(4)	11.621(2)	11.7383(9)	16.267(3)
*c*/Å	12.1942(3)	12.1882(17)	12.3077(10)	16.890(3)
*β/º*				96.578(19)
*V*/Å^3^	1364.77(7)	1369.0(4)	1412.57(19)	1533.5(5)
*Z*	4	4	4	4
Calc. density/mg cm^−3^	1.668	1.663	2.030	1.279
μ/mm^−1^	1.99	1.98	8.79	0.09
*F*(000)	700	700	844	624
θ range/°	2.4–28.5	2.4–28.3	5.2–78.0	2.7–26.7
*R*_int_	0.028	0.031	0.023	0.100
*R*(*F*_obs_)	0.024	0.049	0.031	0.118
*R*_w_(*F*^2^)	0.054	0.085	0.080	0.1765
*S*	1.02	1.06	1.07	1.00
Abs. correction	analytical	semi-empirical	semi-empirical	analytical
Measured reflections	9024	3605	6038	10,253
Independent reflections	3099	2700	2528	5896
Reflections with *I* > 2*σ*(*I*)	2891	2398	2515	2251
Parameters	163	163	164	407

### Synthesis of 2-ethyl-2-((pyridin-2-yl methylene)amino)propane-1,3-diol (HL)

2-Amino-2-ethyl-1,3-propanediol (0.476 g, 4 mmol), pyridine-2-carbaldehyde (0.38 mL, 4 mmol), and methanol (10 mL) were added to a flask. Then, the reaction mixture was stirred at 40 °C for 12 h (Note: prolonged reaction time or higher temperatures will result in the formation of by-products in the synthesis). The reaction mixture was concentrated in a vacuum and the resulting solid was isolated by filtration, which was washed with cold methanol and dried at ambient conditions. Yield: 72%. *Anal*. Calc. for C_11_H_16_N_2_O_2_ (MW = 208.26 g/mol): C, 63.44; H, 7.74; N, 13.45%. Found: C, 63.37; H, 7.71; N, 13.51%. FT-IR (KBr, cm^-1^): 3455 (br, s), 3096 (w), 3093 (w), 3068 (w), 2960 (m), 2958 (m), 2919 (m), 2856 (m), 2803 (m), 1657 (s), 1593 (s), 1573 (s), 1477 (s), 1440 (m), 1381 (w), 1346 (w), 1300 (w), 1227 (m), 1150 (w), 1051 (m), 1035 (m), 1024 (w), 999 (m), 972 (m), 969 (w), 958 (m), 938 (m), 884 (m), 773 (s), 750 (s), 694 (m), 660 (w), 647 (w), 619 (m), 422 (w), 405 (w). ^1^H NMR (250 MHz,CDCl_3_, TMS): δ = 8.51 (s, 1H), 8.11 (s, 1H), 7.65 (s, 1H), 7.39 (s, 1H), 7.22 (s, 1H), 4.96 (s, 2H,OH), 3.62 (s, 2H, CH_2_), 3.54 (s, 2H, CH_2_), 1.57 (s, 2H, CH_2_), 0.77 (s, 3H, CH_3_) ppm. ^13^C NMR (62.9 MHz,CDCl_3_, 25 °C): δ = 157.7, 156.4, 149.1, 137.0, 123.8, 122.2, 71.9, 63.7, 63.4, 27.5 and 7.3 ppm. UV–Vis in CH_3_OH (c = 2 × 10^–5^ M, ε [M^−1^ cm^−1^]): 212 (48,765), 258 nm (45,436).

### Synthesis of [Cu(HL)Cl_2_] (1)

Pro-chiral ligand HL (2.0 mmol, 0.416 g), methanol (15.0 mL), and CuCl_2_·2H_2_O (2.0 mmol, 0.24 g) were added to a 25 mL flask. Then, the resulting solution was stirred at room temperature for half an hour and then, it was refluxed for 6 h. The resulting green solution was cooled to room temperature and allowed to slowly evaporate. The emerald-green crystals were obtained by slow evaporating the solvent for 5 days. The resulting crystals were separated by filtration and washed with cold methanol. Single crystal X-ray diffraction showed the formation of chiral single crystals which indicated conglomerate (two enantiomers) crystal formation during the synthesis and crystallization process. In the following, conglomerate crystals screening were performed by a preferential crystallization method using a single crystal from the initial mother crystal originating. The optical purity of both copper enantiomers was obtained as > 78% ee by HPLC analysis on a chiral stationary phase using methanol/acetonitrile 90:10 solvent (HPLC analysis confirmed that S-[Cu(HL)Cl_2_] enantiopurity is about 86% ee and R-[Cu(HL)Cl_2_] enantiopurity is about 78% ee). Yield of synthesis based on starting ligand: 0.610 g (89%). *Anal*. Calc. for C_11_H_16_Cl_2_CuN_2_O_2_ (MW = 342.70 g/mol): C, 38.55; H, 4.71; N, 8.17; Cu, 18.54%. Found: C, 38.38; H, 4.48; N, 8.25; Cu, 18.59%. FT-IR (KBr, cm^-1^): 3316 (m), 3200 (m), 3104 (w), 3078 (w), 2943 (w), 2883 (w), 1648 (s), 1632 (w), 1603 (s), 1479 (m), 1470 (w), 1462 (s), 1445 (s), 1425 (w), 1398 (m), 1375 (w), 1356 (w), 1287 (s), 1219 (m), 1183 (m), 1124 (m), 1105 (m), 1071 (m), 1062 (m), 1048 (m), 1039 (s), 1024 (m), 1015 (m), 993 (m), 971 (m), 959 (m), 878 (m), 865 (m), 798 (m), 772 (s), 741 (s), 723 (m), 668 (s), 655 (s), 647 (m), 640 (m), 633 (w), 620 (m), 550 (w), 507 (w), 500 (m), 485 (m), 421 (w). UV–Vis in CH_3_OH (c = 2 × 10^–5^ M, ε [M^-1^ cm^-1^]): 234 (54,820), 254 (55,500), 292 nm (54,252).

### Synthesis of [Cu(HL)Br_2_] (2)

S-[Cu(HL)Br_2_] was synthesized using same method as described for **1**. To synthesize complex **2**, HL (2.0 mmol, 0.416 g) was dissolved in methanol and CuBr_2_ (2.0 mmol, 0.446 g) was added to the solution. The mixture was stirred at RT and then, it was refluxed for 6 h. The green crystals of **2** were achieved by evaporating of the resulting green solution at 15–20 °C for a week. Yield: 0.74 g (86%). *Anal*. Calc. for C_11_H_16_Br_2_CuN_2_O_2_ (MW = 431.61 g/mol): C, 30.61; H, 3.74; N, 6.49; Cu, 14.72%. Found: C, 30.55; H, 3.70; N, 6.53; Cu, 14.77%. FT-IR (KBr, cm^-1^): 3436 (m) 3146 (m), 2968 (m), 2949 (m), 2885 (w), 2857 (w), 1646 (m), 1602 (m), 1568 (m), 1466 (m), 1445 (s), 1426 (m), 1397 (m), 1373 (m), 1354 (m), 1325 (s), 1301 (m), 1288 (m), 1256 (m), 1217 (m), 1175 (w), 1152 (w), 1120 (m), 1104 (w), 1088 (w), 1058 (m), 1036 (s), 1023 (m), 1011 (w), 985 (w), 968 (w), 958 (m), 795 (w), 772 (s), 735 (m), 653 (w), 638 (m), 553 (w), 505 (m), 484 (m), 421 (w). UV–Vis in CH_3_OH (c = 2 × 10^–5^ M, ε [M^-1^ cm^-1^]): 230 (51,800), 294 (59,480), 303 (56,800), 402 nm (3092).

### Catalytic synthesis of 1-phenoxy-3-(4-phenyl-1H-1,2,3-triazol-1-yl)propan-2-ol

A flask was charged with chiral Cu(II) complex (1.0–5.0 mol%), phenyl acetylene (1.0 mmol, 0.1 mL), phenyl glycidyl ether (1.0 mmol, 0.13 mL), sodium azide (1.0 mmol, 0.065 g) and water (2.0 mL). The reaction mixture was stirred at RT and upon completion of the reaction (monitored by TLC), the product was separated by solvent extraction process (ethyl acetate 5 × 3 mL) and the crude mixture was purified by column chromatography (ethyl acetate/n-hexane = 2:10). White solid product was recrystallized in ethyl acetate to afford the desired product. The ee value was determined by HPLC. Yield: 0.27 g (91.4%). M.p. 128–130 °C. *Anal*. Calc. for C_17_H_17_N_3_O_2_ (MW = 295.34): C, 69.14; H, 5.80; N, 14.23%. Found: C, 69.05; H, 5.71; N, 14.30%. FT-IR (KBr, cm^−1^): 3556 (w), 3432 (m), 3114 (m), 3087 (m), 3056 (w), 3038 (w), 2938 (m), 2919 (m), 2873 (w), 1599 (s), 1586 (s), 1558 (m), 1498 (s), 1484 (s), 1474 (m), 1465 (s), 1444 (m), 1423 (m), 1378 (m), 1360 (w), 1303 (w), 1291 (m), 1251 (m), 1237 (s), 1175 (m), 1161 (w), 1149 (s), 1123 (w), 1079 (s), 1043 (s), 1021 (w), 977 (s), 913 (m), 878 (w), 836 (m), 817 (s), 764 (vs), 752 (m), 712 (m), 692 (s), 613 (m), 597 (w), 520 (w), 511 (m), 458 (w), 446 (w), 419 (w), 405 (w). ^1^H NMR (250 MHz,CDCl_3_, TMS):δ = 7.85 (s, 1H), 7.71 (d, J = 6.75 Hz, 2H, Ar–H), 7.38–6.90 (m, 8H, Ar–H), 4.71 (m, 1H), 4.54 (d, J = 9.25 Hz, 2H), 4.04 (d, 2H), 3.85 (s, 1H). ^13^C NMR(62.90 MHz, CDCl_3_): δ = 158.1,147.8,130.2, 129.6,128.8,128.2, 125.6,121.6,121.3, 114.5, 68.9, 68.8, 53.1 ppm.

## Results and discussion

### Synthesis and spectroscopic studies

**Figure 2:**
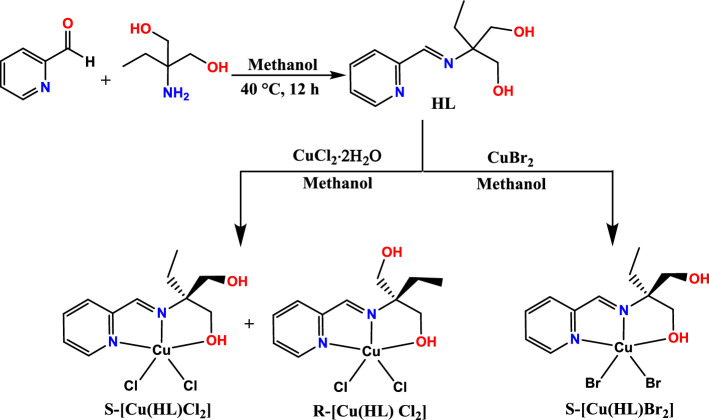
Synthetic pathway and structure of pro-chiral HL ligand and chiral Cu(II) complexes.

The new pro-chiral Schiff base ligand, (2-ethyl-2-((pyridin-2-yl methylene)amino) propane-1,3-diol (HL), was synthesized by the condensation of 2-amino-2-ethyl-1,3-propanediol and 2-pyridinecarbaldehyde in equimolar ratio at 40 °C (see Fig. 2). It should be noted that the selective formation of Schiff base depends on the reaction temperature and also the ratio of reagents. According to our previous reports 1,3-oxazolidine^56^ or bis-oxazole^57^ ligands can be obtained at higher temperatures by further reaction of one or both of the alcoholic arms, respectively. The results of ^1^H NMR, ^13^C NMR, UV–Vis, and FT-IR spectra confirmed the successful synthesis of the ligand. In the infrared spectrum of HL (Fig. S1), the broad characteristic band at 3455 cm^−1^ can be assigned to the –OH groups of the two alcoholic arms^58–60^. The imine bond vibration (υC=N) is observed as a strong band at 1657 cm^−1^ which confirms the successful formation of the Schiff base ligand^61–63^ In the ^1^H NMR spectrum of HL (see Fig. S2), the singlet peak at 8.11 ppm corresponds to the imine group, which can confirm the presence of CH=N group in the structure of this compound. Furthermore, the signals of aromatic protons (pyridine ring) are observed as four peaks at δ 7.22 to 8.51 ppm. The signals of –CH_2_– groups related to the alcoholic arms (–CH_2_OH) appeared at 3.62 and 3.54 ppm. The peaks at 1.57 ppm and 0.88 ppm are related to the –CH_2_– and –CH_3_ groups of the ethyl arm, respectively. Finally, the broad peak at 4.96 ppm is related to OH group of the alcoholic functionalities. In the ^13^C NMR spectrum (Fig. S3), eleven independent peaks are observed that are consistent with the expected ligand structure, providing convincing evidence for its successful synthesis. In this spectrum, the peaks at 27.5 and 7.3 ppm are assigned to the –CH_2_– and –CH_3_ species of the ethyl group, and the peaks at 63.7 and 63.4 ppm can be attributed to the –CH_2_– group of the alcoholic arms. The peak at 71.9 ppm is related to the carbon bonded to nitrogen and the peaks of aromatic carbons together with imine carbon are observed as six peaks in the range of 157.7–122.2 ppm.

**Figure 3:**
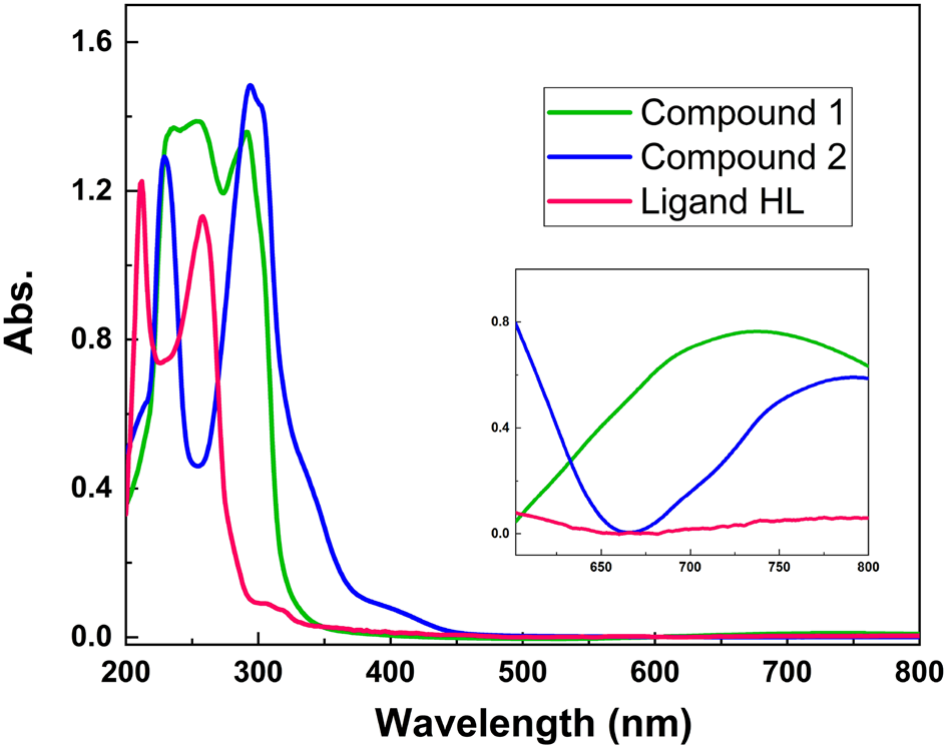
The UV–Vis spectra of the free ligand (HL) and complexes **1** and **2.**

The Cu(II) complexes were obtained by the reaction of HL with copper(II) chloride dihydrate or copper(II) bromide in methanol (see Fig. 2). The crystals of these compounds were obtained by solvent evaporating and the crystals were characterized by several analytical methods. The FT-IR spectra of complexes **1** and **2** are shown in Figs. S4 and S5, respectively. Upon comparison of these spectra with free ligand, it was found that the band assigned to the vibration of imine groups in HL ligand is shifted to lower wavenumbers in both complexes (1648 cm^−1^ in **1** and 1646 cm^−1^ in **2**), denoting coordination of imine nitrogen to Cu(II) ion^64,65^. Also, the change of position and the intensities of the bands in the spectra of **1** and **2** in comparison with the free ligand confirmed its coordination with Cu(II) ion. The UV–Vis absorption spectra of HL, **1** and **2**, recorded in CH_3_OH, are shown in Fig. 3. The spectrum of ligand is composed of two major absorption bands at λ_max_ = 212 and 258 nm which can be attributed to π → π* and n → π* transitions, respectively. After the coordination of ligand to metal, the intraligand absorption band at 212 nm is shifted to 234 nm in complex **1** and 228 in complex **2**. In addition, the band related to intraligand n → π* transition is eliminated in the spectra of complexes which confirms the coordination of ligand through non-bonding electrons of nitrogen atoms. The new absorption bands observed at longer wavelengths in the spectra of **1** and **2** are due to LMCT transitions, indicating the coordination of HL and halide anions (X = Cl, Br).

### Crystal structure of [Cu(HL)Cl_2_] (1)

**Figure 4:**
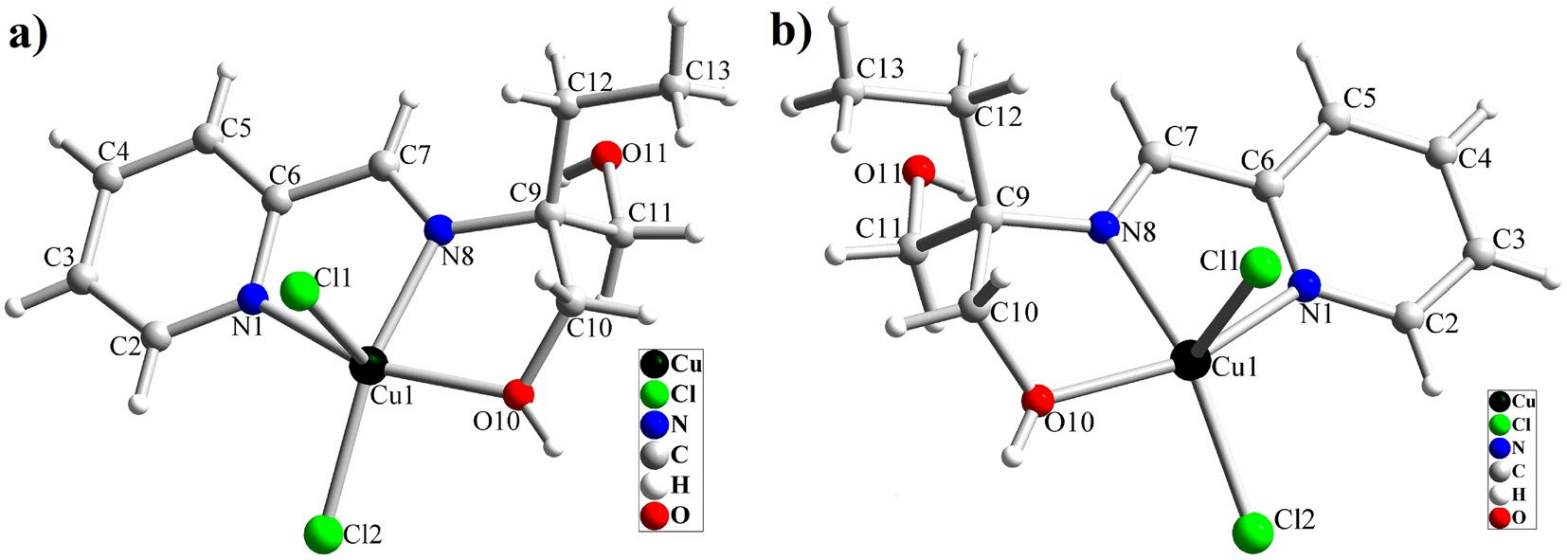
Molecular structure of [Cu(HL)Cl_2_] (**1**); a) R-enantiomer and b) S-enantiomer.

**Figure 5:**
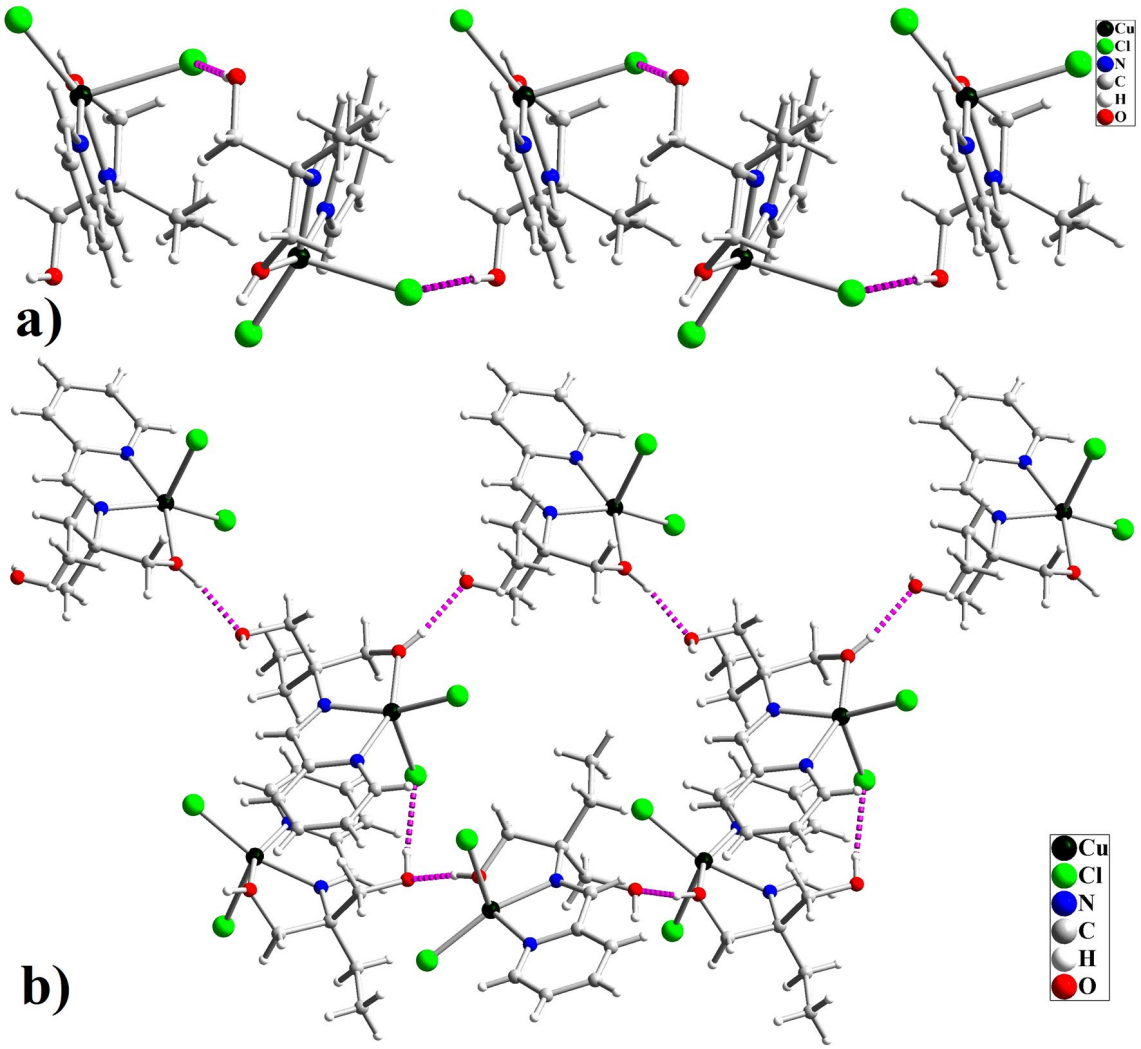
Intermolecular a) O–H···Cl and b) O–H···O interactions in the crystal of S-[Cu(HL)Cl_2_].

One piece of high-quality green crystals of [Cu(HL)Cl_2_], obtained by evaporating the solvent, was selected and it was investigated by single crystal X-ray analysis. X-ray diffraction studies indicated this compound is crystallized in the chiral *P2*_*1*_*2*_*1*_*2*_*1*_ space group of the orthorhombic system. Structural refinement indicated that in the isolated crystal, only one enantiomer (S) exists in the asymmetric unit which means the conglomerate crystallization process has occurred and two enantiomers have been isolated during the formation of crystals. The structure of complex **1** is shown in Fig. 4. This figure indicates the chirality of the complex results from the carbon atom that changes into the stereogenic center after coordinating ligand to the Cu(II) ion. Although HL has two alcoholic arms with coordination capability to metal ions, only one of the alcoholic arms is coordinated to the Cu(II) ion in **1** and as a result, the carbon atom attached to the alcoholic arms is converted to a chiral center. Therefore, the starting ligand is achiral but, it has a high potential to convert to a chiral ligand after coordination to the metal core. Thus, this ligand can be considered as pro-chiral ligand that is not chiral as the free organic compound but, it can form a chiral complex when one of the alcoholic arms is involved in coordination. Since the isolated crystal was S enantiomer of the compound (Fig. 4a), we considered some other pieces of the crystals and found the crystal with R configuration (Fig. 4b). This matter confirmed the production of both enantiomers during the formation of the complex and their isolation through conglomerate crystallization. Both S and R crystals have the same unit cell parameters and the structure of the compound in both cases is the same except for the orientation of four groups connected to the chiral carbon center (C9). The Flack parameter for the S and R crystals is 0.041(19) and 0.002(7), respectively. The near-zero values of the Flack parameter in these structures indicate that the absolute structure determined by structure refinement is correct and these crystals are not racemic or twinning. Compound **1** is a neutral mononuclear Cu(II) complex in which the Cu(II) ion is coordinated by three donor atoms (NNO) of pro-chiral ligand and two chloride anions, forming a slightly distorted square pyramidal geometry around Cu(II) core. The τ parameter is 0.04 and 0.038 for S-[Cu(HL)Cl_2_] and R-[Cu(HL)Cl_2_], respectively which is closer to square pyramidal (τ = 0 for square pyramidal and τ = 1 for trigonal bipyramidal geometry). The three donor atoms of the ligand (N pyridin, N imine, O alcoholic) occupy three corners of the equatorial plane, and one of the chloride anions occupies the fourth equatorial position. The second chloride anion occupies the axial position and the distance between Cu and this chloride anion in S and R molecules is 2.5214(8) and 2.5341(15) Å, respectively. The axial Cu–Cl bond length is considerably longer than other bonds which confirms the presence of Z-out Jahn–Teller distortion along the axial position and is the characteristic for the Cu(II) complexes^66,67^. Table 2 shows the selected bond lengths and angles in the structure of both R and S enantiomers of complex **1**. The Cl1–Cu1–Cl2 angle is 108.37(3)° and 108.56(6)° in S- and R-molecules, respectively. The alcoholic arm of the ligand is coordinated to metal ion without deprotonation and as a result, the ligand acts as a neutral tridentate ligand. The hydrogen atom of this coordinated alcoholic group is involved in intermolecular hydrogen bond interaction with the oxygen atom of the uncoordinated –CH_2_OH group. The OH group of the uncoordinated –CH_2_OH group creates hydrogen bond interaction with the axial chloride ligand in the neighboring molecule. The intermolecular O–H···O, O–H···Cl, C–H···Cl, and also C–H···π interactions stabilize the crystals of this complex. The details of these interactions are collected in Table S1 and Fig. 5 shows a part of these interactions. The intermolecular hydrogen bond interactions connect the molecules to form a 3D network in the solid state.

**Table 2:** Selected bond lengths (Å) and angles (°) in the structure of **1** and** 2.**

Bond	S-[Cu(HL)Cl_2_] (1)	R-[Cu(HL)Cl_2_] (1)	S-[Cu(HL)Br_2_] (2)	
	Length/Å		Bond	Length/Å
Cu1‒N8	1.980 (2)	1.982 (4)	Cu1‒N8	1.978 (5)
Cu1‒O10	2.001 (2)	2.001 (4)	Cu1‒O10	2.005 (4)
Cu1‒N1	2.008 (2)	1.998 (4)	Cu1‒N1	2.017 (5)
Cu1‒Cl1	2.5214 (8)	2.5341 (15)	Cu1‒Br1	2.3603 (10)
Cu1‒Cl2	2.2142 (7)	2.2147 (15)	Cu1‒Br2	2.6606 (10)

Angle	deg/°		Angle	deg/°
N8‒Cu1‒O10	78.26 (8)	78.58 (17)	N8‒Cu1‒O10	78.59 (19)
N8‒Cu1‒N1	81.12 (9)	80.69 (18)	N8‒Cu1‒N1	81.2 (2)
O10‒Cu1‒N1	156.50 (8)	156.49 (17)	O10‒Cu1‒N1	156.68 (19)
N8‒Cu1‒Cl2	158.90 (6)	158.80 (13)	N8‒Cu1‒Br2	160.50 (16)
O10‒Cu1‒Cl2	94.87 (6)	94.68 (12)	O10‒Cu1‒Br2	94.81 (12)
N1‒Cu1‒Cl2	100.42 (6)	100.74 (14)	N1‒Cu1‒Br2	100.47 (14)
N8‒Cu1‒Cl1	92.46 (6)	92.38 (13)	N8‒Cu1‒Br1	91.98 (15)
O10‒Cu1‒Cl1	98.49 (7)	98.48 (13)	O10‒Cu1‒Br1	97.99 (13)
N1‒Cu1‒Cl1	93.52 (6)	93.34 (12)	N1‒Cu1‒Br1	94.22 (13)
Cl2‒Cu1‒Cl1	108.37 (3)	108.56 (6)	Br1‒Cu1‒Br2	107.18 (4)

### Crystal structure of [Cu(HL)Br_2_] (2)

**Figure 6:**
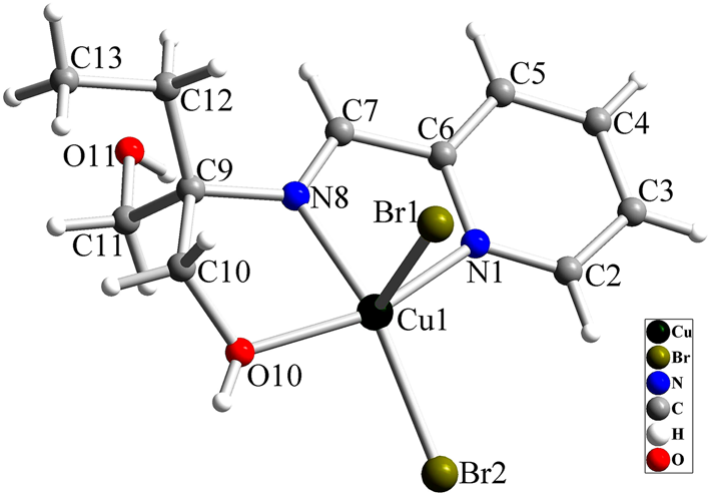
Crystal structure of S-[Cu(HL)Br_2_] (**2**).

By considering the chirality of the crystals in complex **1**, we are interested to use CuBr_2_ as the metal salt in preparing Cu(II) complex and investigate the behavior of ligand in the presence of this Cu(II) salt. Structural studies indicated that **2** has a similar structure to **1**, except Br anion is coordinated to Cu(II) ion in this case (see Fig. 6). The crystals of **2** are also chiral and they are also crystallized in *P2*_*1*_*2*_*1*_*2*_*1*_ space group of the orthorhombic system with approximately the same unit cell parameters. The unit cell parameters (a, b, and c axis) of **2** are slightly larger than **1** which can be related to the presence of Br anion instead of Cl in the structure and its larger atomic radius. X-ray analysis indicated that the absolute configuration in the investigated crystal is S and the Flack parameter is -0.01(2) which confirms the presence of one enantiomer in the crystal. Similar to **1**, the Cu(II) ion is five coordinated in **2** and the CuN_2_OBr_2_ coordination environment has distorted square pyramidal geometry with τ = 0.064. The Cu–N and Cu–O bond lengths in the equatorial positions of **2** are close to the similar bonds in **1** (see Table 2) and are in the normal range reported in similar Cu(II) complexes^68–71^. The Cu–Br1 and Cu–Br2 bond lengths are 2.3602(10) and 2.6607(10) Å, respectively and confirm their coordination to equatorial and axial positions and the presence of Jahn–Teller distortion in the axial direction. The Br1–Cu1–Br2 in S-[Cu(HL)Br_2_] is 107.18(4)°. The intermolecular interactions in the crystal of **2** are also similar to **1** and the crystal is stabilized by O–H···O, O–H···Br, C–H···Br and C–H···π interactions (Fig. S7 and Table S3).

### CD chiroptical properties

**Figure 7:**
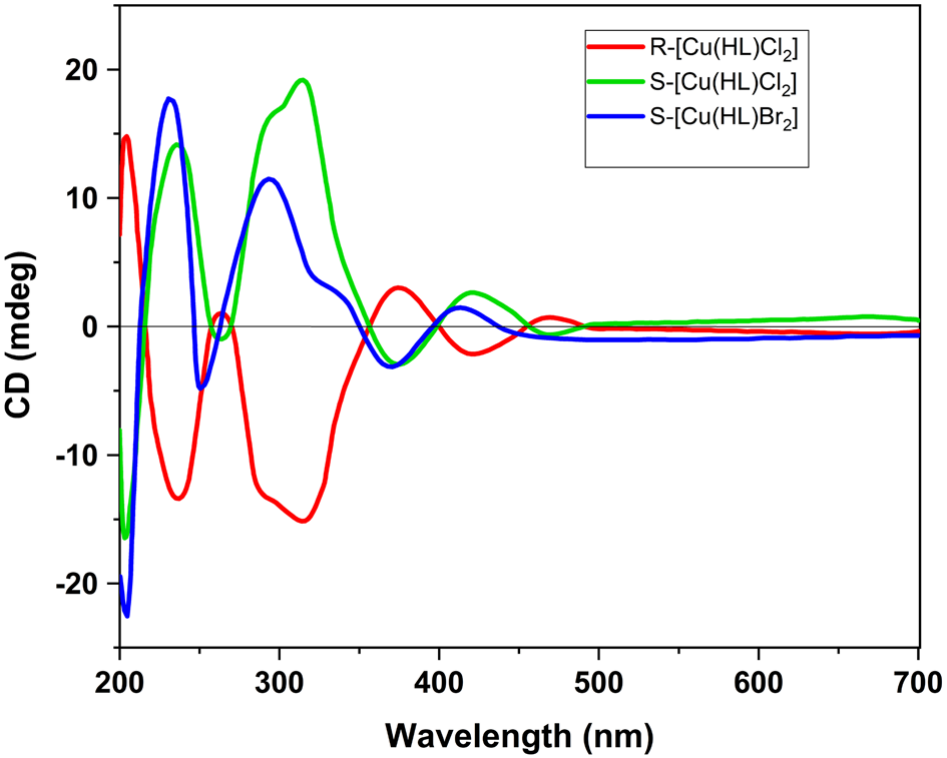
CD spectra of **1** and **2** recorded in CH_3_OH solution (1 × 10^–5^ M) at 20 °C.

By considering the formation of chiral complex and the isolation of R and S isomers in the crystallization process, we are interested in investigating them by circular dichroism (CD) spectroscopy. Thus, preferential crystallization was used to isolate the R and S enantiomers in the crystalline format, and after preferential crystallization, the S and R enantiomers of [Cu(HL)Cl_2_] were isolated with about 86% ee and 78% ee, respectively (see Figs. S8 and S9). Also, during the six-day solvent evaporation crystallization process, the S enantiomer of [Cu(HL)Br_2_] was produced as the main crystal. After several attempts, we could not isolate the R enantiomer of this compound as high-quality single crystal for X-ray diffraction studies. This matter may be related to the formation of low amounts of this enantiomer in the applied crystallization condition. The chiroptical properties of enantiomers in these compounds were studied by CD spectroscopy in methanol solvent at ≈ 20 °C and the results are shown in Fig. 7. CD spectra were also recorded at 0 and 40 °C and the resulting spectra were the same as the spectra obtained at 20 °C which indicate changing temperature (± 20 °C) does not have considerable effect on the CD spectra of these samples. The result shows that [Cu(HL)Br_2_] has a total spontaneous resolution. Although the enantiomers have little variations in the CD spectra, the predicted mirror-image relationship between R-[Cu(HL)Cl_2_] and S-[Cu(HL)Cl_2_] can be seen in the electronic CD spectra of this compound. The R-[Cu(HL)Cl_2_] molecule shows the following series of CD bands (sign, and strength): 466 (+, weak); 421 (−, weak); 373 (+, medium); 317 (−, strong); 265 (+, weak); 237 (−, medium); 207 nm (+, strong). The CD spectra of the S-molecule give mirror images of the R-[Cu(HL)Cl_2_] and seven signals are observable between 200 and 700 nm: 467 (−, weak); 419 (+, weak); 372 (−, medium); 315 (+, strong); 262 (−, weak); 240 (+, medium); 208 nm (−, strong). S-[Cu(HL)Br_2_] shows the following series of CD bands which indicate its S configuration: 413 (+, weak); 372 (−, weak); 334 (+, weak); 294 (+, medium); 250 (−, medium); 224 (+, strong) and 205 nm (−, strong).

### Catalytic synthesis of chiral 1,2,3-triazole by 1 and 2

**Figure 8:**
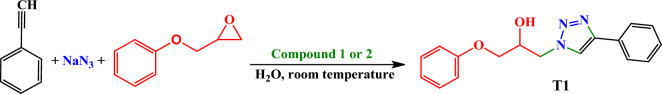
Catalytic synthesis of 1-phenoxy-3-(4-phenyl-1H-1,2,3-triazol-1-yl)propan-2-ol (**T1**) in the presence of **1** or **2.**

**Figure 9:**
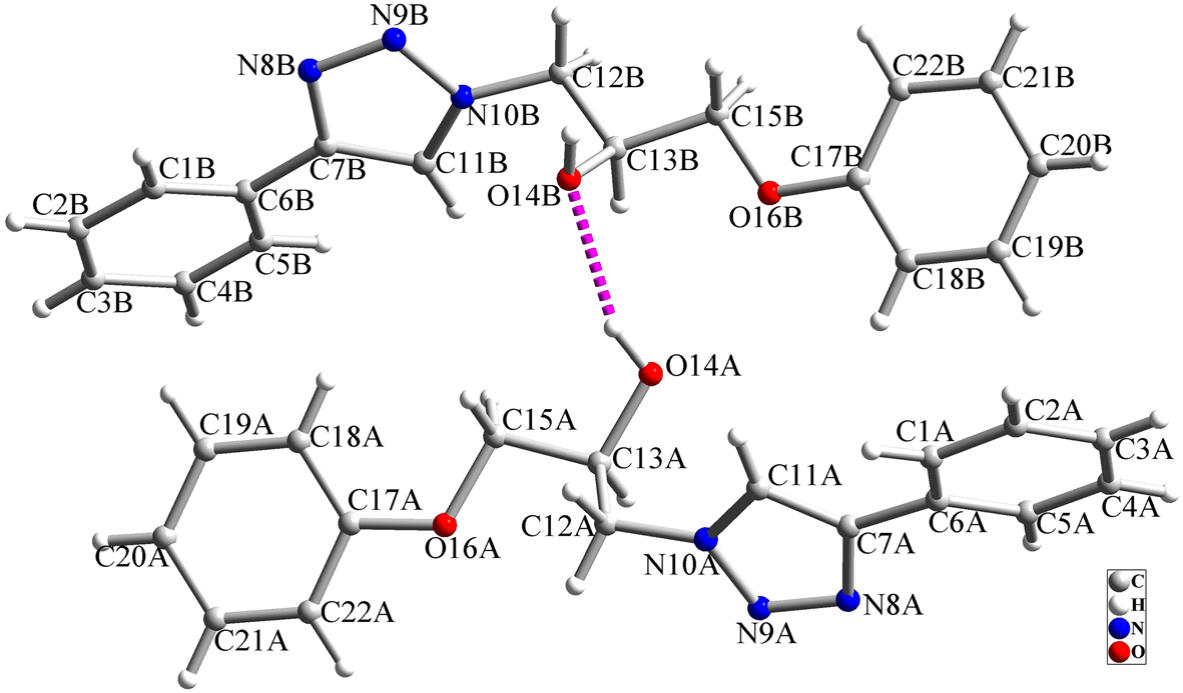
Molecular structure of triazole product (**T1**).

In recent years, the development of chiral catalytic systems for asymmetric synthesis has been an attractive research area, and due to this, the complexes containing chiral and pro-chiral ligands have also received significant attention. The synthesis of chiral homogeneous metal catalysts offers distinct advantages like selectivity, higher activity, and easy accessibility of the catalytically active sites. Therefore, we are interested to study the catalytic performance of the obtained chiral complexes in the synthesis of β-hydroxy-1,2,3-triazole by using epoxide-azide-alkyne cycloaddition reaction. The resulting β-hydroxy-1,2,3-triazoles through this reaction have a chiral center and they can be obtained as chiral products by controlling the epoxide ring opening reaction. For the investigation of this objective, the catalytic synthesis of a triazole compound by the reaction of phenylacetylene, sodium azide, and a racemic mixture of phenyl glycidyl ether was examined as a model reaction (see Fig. 8). The catalytic syntheses were done in water as the green solvent in the presence of S-[Cu(HL)Cl_2_] (3.0 mol%) as the catalyst. The results of the catalytic tests at different reaction conditions are summarized in Table 3. The results showed that S-[Cu(HL)Cl_2_] can efficiently catalyze the formation of target triazole at room temperature. The structure of the triazole product was investigated by X-ray diffraction studies and the ratio of enantioselectivity was obtained by chiral stationary phase HPLC. Although due to the absence of a heavy atom in the structure of the triazole product, it is not possible to determine its absolute configuration at the stereogenic carbon center, the crystals of this product are also crystallized in the chiral *P2*_*1*_ space group (monoclinic system). It must be noted that in this crystal, according to the observed electron densities, approximately 10% of the racemic mixture can be observed. This matter confirms that the main part of the isolated product is chiral and one enantiomer is considerably excess than the other and as a result, the compound is crystallized in the chiral format. This matter is also in agreement with the results of catalytic reactions and the observed enantioselectivity in the catalytic reactions. The structure of the product is shown in Fig. 9 and selected structural parameters are given in Table S4. Structural studies indicate that the product is obtained by the ring opening reaction of aliphatic epoxide via its less substituted carbon atom which is also in agreement with previous reports in the literature^72–75^. The crystal of the product is stabilized by intermolecular interactions which are shown in Fig. S10 and Table S4. The ^1^H and ^13^C NMR spectra of the product are shown in Figs. S11 and S12 which are in agreement with its structure.

**Table 3:** Optimization of catalyst for the synthesis of 1,2,3-triazole (**T1**).

Entry	Catalyst	Mol%	Time (h)	Temp. (°C)	Yield (%)	ee%
1	S-[Cu(HL)Cl_2_]	1	6	25	21	40
2	S-[Cu(HL)Cl_2_]	3	6	25	60	34
3	S-[Cu(HL)Cl_2_]	5	6	25	64	32
4	S-[Cu(HL)Cl_2_]	3	10	25	96	0
5	S-[Cu(HL)Cl_2_]	3	6	50	72	16
6	S-[Cu(HL)Cl_2_]	3	6	70	87	3
7	S-[Cu(HL)Cl_2_]	3	6	90	98	0
8	R-[Cu(HL)Cl_2_]	3	6	25	59	29 (R)
9	S-[Cu(HL)Br_2_]	3	6	25	65	35
10	S-[Cu(HL)Br_2_]	3	6	50	76	18
11	S-[Cu(HL)Br_2_]	3	6	70	88	6
12	S-[Cu(HL)Br_2_]	3	6	90	98	0

Considering the reaction mixture by chiral HPLC indicated that S-enantiomer of the triazole product was higher than the R-enantiomer up to 6 h (yield ≈ 60%, ee% ≈ 34) and after that, the enantioselectivity was continuously decreased. This matter indicates the S-enantiomer of epoxide reacts faster than R enantiomer in the presence of S-[Cu(HL)Cl_2_]. By decreasing the ratio of S to R enantiomers of epoxide in the reaction mixture, the R enantiomer also involved in the reaction. If the reaction continues to finish the entire epoxide reagent, a racemic mixture will be obtained. Thus, although both of the epoxide enantiomers can be involved in the cyclization reaction, S-[Cu(HL)Cl_2_] prefers to use S-enantiomer of epoxide rather than R-enantiomer. In the next step, the reactions were done at 50, 70, and 90 °C to investigate the effect of temperature. The results showed that enantioselectivity considerably decreases by increasing the temperature of the reaction. At 70 °C almost a racemic mixture was obtained which indicates at higher temperatures both enantiomers of the epoxide can simultaneously be involved in the reaction and there is no considerable difference in their reactivity in the presence of the employed chiral catalysts. At 90 °C a byproduct was also obtained by the ring opening reaction via more substituted carbon of the epoxide reagent. By using R-[Cu(HL)Cl_2_] similar results were obtained but, the ee % in this case was slightly lower.

*Reaction condition*: Phenylacetylene (1.0 mmol), phenyl glycidyl ether (1.0 mmol), azide (1.1 mmol), water (5.0 mL).

For further investigations, the synthesis of 1,2,3-triazole was carried out using complex **2**. The yield and enantioselectivity of the product were slightly improved in the presence of compound **2**. Comparing the results indicates that compound **2** has higher catalytic activity than compound **1**. This matter is most likely due to the differences of the halide anions in the structure of these compounds and the higher leaving ability of the bromide anion in comparison with chloride. The results of catalytic studies indicate that these chiral complexes can act as appropriate catalysts for the asymmetric synthesis of β-hydroxy-1,2,3-triazole from a racemic mixture of epoxides under controlled conditions. Both **1** and **2** afford acceptable conversions of epoxy, acetylene, and azide to the 1,2,3-triazole under mild and green conditions.

**Figure 10:**
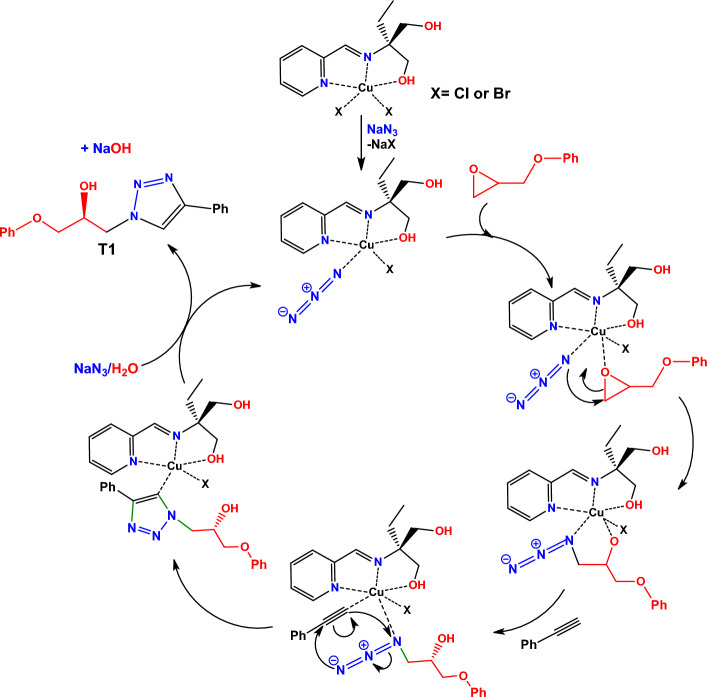
Suggested mechanism for the synthesis of **T1** in the presence of **1** and **2.**

The mechanism of the 1,2,3-triazole formation in the presence of Cu(II) complexes has been widely investigated in the literature^76–82^. Based on earlier reports, a plausible mechanism was presented for this reaction which is shown in Fig. 10. As is demonstrated, in the first step the azide and epoxide are coordinated to the Cu(II) core. The azide, as a nucleophile, attacks the less substituted carbon of the epoxide ring in phenyl glycidyl ether to form alkyl-azide intermediate. In the presence of a chiral complex, this ring opening reaction is the most important process in the formation of chiral 1,2,3-triazole by considering the fact that the chiral center in the triazole generates in this step. The uncoordinated alcoholic functionality of the ligand in **1** and **2** can have a considerable effect on the formation of this intermediate by the formation of hydrogen bond interactions with the oxygen atoms of the employed epoxide. Such interactions together with steric hindrance around the chiral center make differences in the activity of two enantiomers of the employed epoxide and one of them reacts faster to make enantioselectivity in the ring opening reaction which causes the formation of chiral crystals of triazole product. In the next step, phenyl acetylene coordinates to the copper core and the cycloaddition reaction of copper(II) acetylide with alkyl-azide intermediate takes place to create a triazole ring through click reaction. Finally, protonolysis of the Cu–C center in the presence of water solvent leads to the formation of the final product and the catalytically active intermediate regenerates by coordination of the next azide ion from sodium azide reagent.

## Conclusion

In summary, the reaction of prochiral ligand (HL) with Cu(II) salts gave mononuclear Cu(II) complexes, [Cu(HL)Cl_2_] (**1**) and [Cu(HL)Br_2_] (**2**). The R- and S- enantiomers of **1** and S-enantiomer of **2** were isolated in crystalline format during the slow solvent evaporation of their methanolic solutions. The crystal structure of S-[Cu(HL)Cl_2_], R-[Cu(HL)Cl_2_], and S-[Cu(HL)Br_2_] were determined by X-ray analysis. The R and S enantiomers were isolated in crystalline format by preferential crystallization method. The HPLC study on the chiral stationary phase also clearly revealed that the conglomerates were satisfactorily separated with preferred crystallization. Structural studies indicated that coordination of ligand to Cu(II) ion converts ligand to a chiral compound. The obtained chiral Cu(II) complexes were used as catalysts in the click synthesis of β-hydroxy-1,2,3-triazole compounds and the results of catalytic studies indicated that **1** and **2** can act as enantioselective catalysts for the asymmetric synthesis of β-hydroxy-1,2,3-triazole product under mild condition.

### Supplementary Information

Below is the link to the electronic supplementary material.

Supplementary Information 1.

Supplementary Information 2.

Supplementary Information 3.

Supplementary Information 4.

Supplementary Information 5.

Supplementary Information 6.

## Data Availability

The supporting information containing spectroscopic data of the compounds, material and methods, details of X-ray analysis and also intermolecular interactions are available in the online version at doi:. The structural data have been deposited at the Cambridge Crystallographic Data Centre (www.ccdc.cam.ac.uk/data_request/cif). CCDC 2288813–2288815 and 1921722 contain crystallographic data for S-(**1**), R-(**1**), R-(**2**), and **T1**, respectively.

## References

[1] Silvestri I, Colbon PJJ (2021). The growing importance of chirality in 3D chemical space exploration and modern drug discovery approaches for hit-ID ACS med. Chem. Lett.

[2] Niu, X. *et al.* Chiral materials: Progress, applications, and prospects. *Small* **19**, 2303059 (2023). 10.1002/smll.202303059 37217989

[3] Mwamwitwa KW (2020). A retrospective cross-sectional study to determine chirality status of registered medicines in Tanzania. Sci. Rep..

[4] Karimi B, Jafari E, Mansouri F, Tavakolian M (2023). Catalytic asymmetric Friedel–Crafts alkylation of unprotected indoles with nitroalkenes using a novel chiral Yb(OTf)3–pybox complex. Sci. Rep..

[5] Zhang Z (2021). Supramolecular asymmetric catalysis mediated by crown ethers and related recognition systems. Green Synth. Cata.

[6] Zhang X, Wang F, Tan C-H (2023). Asymmetric synthesis of S (IV) and S (VI) stereogenic centers. JACS Au.

[7] Bai Y-Q (2023). Design and synthesis of planar-chiral oxazole-pyridine N,N-ligands: Application in palladium-catalyzed asymmetric acetoxylative cyclization. ACS Catal..

[8] Yasukawa, T. & Kobayashi, S. Chiral metal nanoparticles for asymmetric catalysis. *Nanopart. Catal.* **66**, 279–314 (2020).

[9] Nagib DA (2022). Asymmetric catalysis in radical chemistry. Chem. Rev..

[10] Parvatkar PT, Smotkin ES, Manetsch R (2021). Total synthesis of (±)-decursivine via BINOL-phosphoric acid catalyzed tandem oxidative cyclization. Sci. Rep..

[11] Łowicki D, Baś S, Mlynarski J (2015). Chiral zinc catalysts for asymmetric synthesis. Tetrahedron.

[12] Wang G, Zhou Z, Shen X, Ivlev S, Meggers E (2020). Asymmetric catalysis with a chiral-at-osmium complex. Chem. Commun..

[13] Dey P, Rai P, Maji B (2021). Recent development of bis-cyclometalated chiral-at-iridium and rhodium complexes for asymmetric catalysis. ACS Org. Inorg. Au.

[14] Liu Z-S (2020). Construction of axial chirality via palladium/chiral norbornene cooperative catalysis. Nature Catal..

[15] Singha S, Buchsteiner M, Bistoni G, Goddard R, Fürstner A (2021). A new ligand design based on London dispersion empowers chiral bismuth–rhodium paddlewheel catalysts. J. Am. Chem. Soc..

[16] Huang Y, Hayashi T (2022). Chiral diene ligands in asymmetric catalysis. Chem. Rev..

[17] Su B, Hartwig JF (2022). Development of chiral ligands for the transition-metal-catalyzed enantioselective silylation and borylation of C–H bonds. Angew. Chem. Int. Ed..

[18] Jiang HJ (2019). Assembling a hybrid Pd catalyst from a chiral anionic CoIII complex and ligand for asymmetric C (sp3)–H functionalization. Angew. Chem. Int. Ed..

[19] Hao J (2020). Ligand-induced chirality in asymmetric CdSe/CdS nanostructures: a close look at chiral tadpoles. ACS Nano.

[20] Yoshizawa A, Feula A, Male L, Leach AG, Fossey JS (2018). Rigid and concave, 2,4-cis-substituted azetidine derivatives: A platform for asymmetric catalysis. Sci. Rep..

[21] Akine S, Miyake H (2022). Stimuli-responsive chirality inversion of metallohelices and related dynamic metal complexes. Coord. Chem. Rev..

[22] Liu, G., Humphrey, M. G., Zhang, C. & Zhao, Y. Self-assembled stereomutation with supramolecular chirality inversion. *Chem. Soc. Rev. ***52**, 4443-4487 (2023). 10.1039/d2cs00476c 37337858

[23] Huang K-L, He Y-T, Liang G-M, Sun Y-Y, Hu C-W (2007). Photoluminescent metal (II)-organic complexes with two distinct atropisomeric units from axially prochiral ligands through C-H O hydrogen bonds (M = zinc, cobalt, nickel). Inorg. Chim. Acta.

[24] S.K. Talapatra, B. Talapatra, prochirality and prostereoisomerism. Topicity of ligands and faces nomenclature [1–5]. In *Basic Concepts in Organic Stereochemistry* 71–86 (Springer, 2023).

[25] Talapatra, S. K. & Talapatra, B. In *Basic Concepts in Organic Stereochemistry* 71–86 (Springer, 2023).

[26] Bernal I, Cetrullo J, Jackson WG (1993). The phenomenon of conglomerate crystallization. XXV. Spontaneous resolution in coordination compounds. The crystal structure of [cis-co (en)_2_ (nh_3_) br] br_2_. J. Coord. Chem..

[27] Kong L (2019). From prochiral N-heterocyclic carbenes to optically pure metal complexes: New opportunities in asymmetric catalysis. J. Am. Chem. Soc..

[28] Rybak WK, Cymbaluk A, Siczek M, Skonieczny J (2012). Crystallization-induced asymmetric synthesis of nonracemic platinum (IV) polysulfide tris (chelate) complexes. Eur. J. Inorg. Chem..

[29] Rybak WK, Skarżyńska A, Głowiak T (2003). Efficient asymmetry generation in the synthesis of oxo-rhenium (V) complex cis-[ReOCl2 OCMe_2_CMe_2_OP (OCMe_2_CMe_2_O) py]. Angew. Chem. Int. Ed..

[30] Kelly CT, Jordan R, Felton S, Müller-Bunz H, Morgan GG (2023). Spontaneous chiral resolution of a MnIII spin-crossover complex with high temperature 80 K hysteresis. Chem. A Eur. J..

[31] Pinètre C (2023). Use of conglomerate mixed crystals to deracemize a stable racemic-compound-forming system. Chem. Eur. J..

[32] Matin MM (2022). Triazoles and their derivatives: Chemistry, synthesis, and therapeutic applications. Front. Mol. Biosci..

[33] Mortazavi M (2023). Novel quinazoline-1,2,3-triazole hybrids with anticancer and MET kinase targeting properties. Sci. Rep..

[34] da SM Forezi L (2021). Bioactive 1, 2, 3-triazoles: An account on their synthesis, structural diversity and biological applications. Chem. Rec..

[35] Vala DP, Vala RM, Patel HM (2022). Versatile synthetic platform for 1, 2, 3-triazole chemistry. Acs Omega.

[36] Rammohan A, Venkatesh BC, Basha NM, Zyryanov GV, Nageswararao M (2023). Comprehensive review on natural pharmacophore tethered 1, 2, 3-triazoles as active pharmaceuticals. Chem Biol. Drug. Des..

[37] Steppeler F (2023). Chiral 2-azabicycloalkanes bearing 1, 2, 3-triazole, thiourea, and ebselen moieties—synthesis and biological activity. Biomed. Pharmacother.

[38] Musarurwa H, Tavengwa NT (2020). Green aspects during synthesis, application and chromatographic analysis of chiral pesticides. Trends Environ. Anal. Chem..

[39] Guo W-T (2022). Enantioselective rh-catalyzed azide-internal-alkyne cycloaddition for the construction of axially chiral 1, 2, 3-triazoles. J. Am. Chem. Soc..

[40] Brittain WDG (2019). Coetaneous catalytic kinetic resolution of alkynes and azides through asymmetric triazole formation. Sci. Rep..

[41] He Q, Zhang D, Zhang F, Liu X, Feng X (2021). Asymmetric catalytic epoxidation of terminal enones for the synthesis of triazole antifungal agents. Org. Lett..

[42] Liu E-C, Topczewski JJ (2019). Enantioselective copper catalyzed alkyne–azide cycloaddition by dynamic kinetic resolution. J. Am. Chem. Soc..

[43] Dhameja M, Kumar H, Gupta P (2020). Chiral fused 1, 2, 3-triazoles: A synthetic overview. Asian J. Org. Chem..

[44] Stålsmeden AS (2020). Chiral 1, 5-disubstituted 1, 2, 3-triazoles–versatile tools for foldamers and peptidomimetic applications. Org. Biomol. Chem..

[45] Bica K, Gaertner P (2008). Applications of chiral ionic liquids. Eur. J. Org. Chem..

[46] Kaur, N., Singh, A., Kaur, P. & Chopra, H. K. In *Ionic Liquids in Analytical Chemistry* 275–296 (Elsevier, 2022).

[47] Wright AJ, Hughes DL, Bulman Page PC, Stephenson GR (2019). Induction of planar chirality using asymmetric click chemistry by a novel desymmetrisation of 1, 3-bisalkynyl ferrocenes. Eur. J. Org. Chem..

[48] Zeng L, Zhang F, Cui S (2023). Construction of axial chirality via click chemistry: Rh-catalyzed enantioselective synthesis of 1-triazolyl-2-naphthylamines. Org. Lett..

[49] Osako T, Uozumi Y (2014). Enantioposition-selective copper-catalyzed azide-alkyne cycloaddition for construction of chiral biaryl derivatives. Org. Lett..

[50] Ghosh D (2020). A Cu(II)-inorganic Co−crystal as a versatile catalyst towards ‘click’ chemistry for synthesis of 1,2,3-triazoles and β-hydroxy-1,2,3-triazoles. ChemistrySelect.

[51] Li J, Ren Y, Qi C, Jiang H (2017). A chiral salen-based MOF catalytic material with high thermal, aqueous and chemical stabilities. DaltonTrans..

[52] Anil Kumar BSP, Harsha Vardhan Reddy K, Satish G, Uday Kumar R, Nageswar YVD (2014). Synthesis of β-hydroxy-1,4-disubstituted-1,2,3-triazoles catalyzed by copper ferrite nanoparticles in tap water using click chemistry. RSC Adv..

[53] Alvarenga N, Porto ALM, Barreiro JC (2018). Enantioselective separation of (±)-β-hydroxy-1,2,3-triazoles by supercritical fluid chromatography and high-performance liquid chromatography. Chirality.

[54] Yang X, Wang H, Jin Z, Chi YR (2021). Development of green and low-cost chiral oxidants for asymmetric catalytic hydroxylation of enals. Green Synth. Catal..

[55] Blaser, H.-U. In *Homogeneous Hydrogenation with Non‐Precious Catalysts* 1–14 (2019).

[56] Bikas R, Ajormal F, Emami M, Noshiranzadeh N, Kozakiewicz A (2018). Catalytic oxidation of benzyl alcohols by new Cu(II) complexes of 1,3-oxazolidine based ligand obtained from a solvent free reaction. Inorg. Chim. Acta.

[57] Ajormal F, Bikas R, Noshiranzadeh N, Kozakiewicz-Piekarz A, Lis T (2022). Green catalytic synthesis of symmetric and non-symmetric β-hydroxy-1,2,3-triazoles by using epichlorohydrin in the presence of Cu(ii) coordination compounds containing oxazole ligands. New J. Chem.

[58] Soltani F, Bikas R, Heydari N, Kozakiewicz-Piekarz A (2023). Dinuclear oxidovanadium complexes with dihydrazone ligands derived from diethyl 2,6-dimethylpyridine-3,5-dicarboxylate obtained from Hantzsch reaction; crystal structure and catalytic activity. New J. Chem..

[59] Noshiranzadeh N, Heidari A, Haghi F, Bikas R, Lis T (2017). Chiral lactic hydrazone derivatives as potential bioactive antibacterial agents: Synthesis, spectroscopic, structural and molecular docking studies. J. Mol. Struct..

[60] Chattopadhyay K, Shaw BK, Saha SK, Ray D (2016). Unique trapping of paddlewheel copper(ii) carboxylate by ligand-bound Cu2 fragments for [Cu6] assembly. Dalton Trans..

[61] Wang D, Wang T, Yang H, Yang J, Shi Z (2023). Spectroscopy and visible-light driven photocatalytic properties of a microcrystalline Cu-complex derived from a novel Gabapentin Schiff base. Spectrochim. Acta A Mol. Biomol..

[62] Rohini R (2011). Synthesis of mono, bis-2-(2-arylideneaminophenyl) indole azomethines as potential antimicrobial agents. Arch. Pharm. Res..

[63] Abou-Hussein AA, Linert W (2014). Synthesis, spectroscopic, coordination and biological activities of some organometallic complexes derived from thio-Schiff base ligands. Spectrochim. Acta A Mol. Biomol..

[64] Côrte-Real L (2023). Cu(II) and Zn(II) complexes of new 8-hydroxyquinoline Schiff bases: Investigating their structure, solution speciation, and anticancer potential. Inorg. Chem..

[65] Ashok UP (2020). Preparation, spectroscopic characterization, theoretical investigations, and in vitro anticancer activity of Cd(II), Ni(II), Zn(II), and Cu(II) complexes of 4(3H)-quinazolinone-derived Schiff base. Molecules.

[66] Conradie J (2019). Jahn–Teller effect in high spin d4 and d9 octahedral metal-complexes. Inorg. Chim. Acta.

[67] Bandopadhyay N (2021). Unprecedented copper(ii) coordination induced nucleophilic cleavage of a quinoxaline heterocycle: structural and computational studies. CrystEngComm.

[68] Bikas R, Ajormal F, Noshiranzadeh N, Emami M, Kozakiewicz A (2020). 1D Azido bridged Cu(II) coordination polymer with 1,3-oxazolidine ligand as an effective catalyst for green click synthesis of 1,2,3-triazoles. Appl. Organomet. Chem..

[69] Bivián-Castro EY (2023). Synthesis and characterization of a new Cu(II) Paddle-Wheel-like complex with 4-vinylbenzoate as an inorganic node for metal–organic framework material design. Materials.

[70] Heydari N, Bikas R, Siczek M, Lis T (2023). Green carbon–carbon homocoupling of terminal alkynes by a silica supported Cu (ii)-hydrazone coordination compound. Dalton Trans..

[71] Ali Akbari MS, Nandy S, Chae KH, Bikas R, Kozakiewicz-Piekarz A, Najafpour MM (2023). Water oxidation by a copper (II) complex with 6, 6′-dihydroxy-2, 2′-bipyridine ligand: Challenges and an alternative mechanism. Langmuir.

[72] Emami M, Bikas R, Noshiranzadeh N, Kozakiewicz A, Lis T (2020). Cu(II)-hydrazide coordination compound supported on silica gel as an efficient and recyclable heterogeneous catalyst for green click synthesis of β-hydroxy-1,2,3-triazoles in water. ACS Omega.

[73] Alonso F, Moglie Y, Radivoy G, Yus M (2011). Multicomponent click synthesis of 1,2,3-triazoles from epoxides in water catalyzed by copper nanoparticles on activated carbon. J. Org. Chem..

[74] Noshiranzadeh N, Emami M, Bikas R, Kozakiewicz A (2017). Green click synthesis of β-hydroxy-1,2,3-triazoles in water in the presence of a Cu(ii)–azide catalyst: a new function for Cu(ii)–azide complexes. New J. Chem..

[75] Lee M, Lamb JR, Sanford MJ, LaPointe AM, Coates GW (2018). Nucleophilic ring opening of trans-2,3-disubstituted epoxides to β-amino alcohols with catalyst-controlled regioselectivity. Chem. Commun..

[76] Meldal M, Tornøe CW (2008). Cu-catalyzed azide−alkyne cycloaddition. Chem. Rev..

[77] González-Lainez M (2022). Copper-catalyzed azide-alkyne cycloaddition (CuAAC) by functionalized NHC-based polynuclear catalysts: Scope and mechanistic insights. Organometallics.

[78] Worrell BT, Malik JA, Fokin VV (2013). Direct evidence of a dinuclear copper intermediate in Cu(I)-catalyzed azide-alkyne cycloadditions. Science.

[79] Sharma C, Kaur M, Choudhary A, Sharma S, Paul S (2020). Nitrogen doped carbon-silica based Cu(0) nanometal catalyst enriched with well-defined n-moieties: synthesis and application in one-pot synthesis of 1,4-disubstituted-1,2,3-triazoles. Catal. Lett..

[80] Bakherad M, Keivanloo A, Amin AH, Farkhondeh A (2019). Synthesis of 1,2,3 triazole-linked benzimidazole through a copper-catalyzed click reaction. Heterocycl. Comm..

[81] Jankovič D, Virant M, Gazvoda M (2022). Copper-catalyzed azide-alkyne cycloaddition of hydrazoic acid formed in situ from sodium azide affords 4-monosubstituted-1,2,3-triazoles. J. Org. Chem..

[82] Alonso F, Moglie Y, Radivoy G, Yus M (2011). Multicomponent click synthesis of 1, 2, 3-triazoles from epoxides in water catalyzed by copper nanoparticles on activated carbon. J. Org. Chem..

