# Supplementary material for: Synthesis of chiral Cu(II) complexes from pro-chiral Schiff base ligand and investigation of their catalytic activity in the asymmetric synthesis of 1,2,3-triazoles
Source: Sci Rep. 2024 May 8;14:10603. doi: 10.1038/s41598-024-60930-w (PMC11079015; doi:10.1038/s41598-024-60930-w)

# checkCIF/PLATON report

Structure factors have been supplied for datablock(s) ft602

THIS REPORT IS FOR GUIDANCE ONLY. IF USED AS PART OF A REVIEW PROCEDURE FOR PUBLICATION, IT SHOULD NOT REPLACE THE EXPERTISE OF AN EXPERIENCED CRYSTALLOGRAPHIC REFEREE.

No syntax errors found. CIF dictionary Interpreting this report

## Datablock: ft602

---

```
Bond precision:   C-C = 0.0042 A          Wavelength=0.71073

Cell:              a=9.6111(3)      b=11.6447(4)      c=12.1942(3)
                   alpha=90         beta=90           gamma=90
Temperature:       293 K

                Calculated                    Reported
Volume          1364.76(7)                    1364.77(7)
Space group     P 21 21 21                    P 21 21 21
Hall group      P 2ac 2ab                     P 2ac 2ab
Moiety formula  C11 H16 Cl2 Cu N2 O2          C11 H16 Cl2 Cu N2 O2
Sum formula     C11 H16 Cl2 Cu N2 O2          C11 H16 Cl2 Cu N2 O2
Mr              342.71                        342.70
Dx,g cm-3       1.668                         1.668
Z               4                             4
Mu (mm-1)       1.986                         1.986
F000            700.0                         700.0
F000'           702.64
h,k,lmax        12,15,16                      12,14,15
Nref            3463[ 1983]                   3099
Tmin,Tmax       0.507,0.748                   0.424,0.772
Tmin'           0.294

Correction method= # Reported T Limits: Tmin=0.424 Tmax=0.772
AbsCorr = ANALYTICAL

Data completeness= 1.56/0.89       Theta(max)= 28.505

R(reflections)= 0.0221( 2891)                          wR2(reflections)=
                                                       0.0544( 3099)
S = 1.019                  Npar= 163
```

The following ALERTS were generated. Each ALERT has the format
**test-name_ALERT_alert-type_alert-level.**
Click on the hyperlinks for more details of the test.

## Alert level C

```
PLAT790_ALERT_4_C Centre of Gravity not Within Unit Cell: Resd.  #          1 Note
              C11 H16 Cl2 Cu N2 O2
```

## Alert level G

```
PLAT007_ALERT_5_G Number of Unrefined Donor-H Atoms .............           2 Report
PLAT063_ALERT_4_G Crystal Size Possibly too Large for Beam Size ..       0.61 mm
PLAT199_ALERT_1_G Reported _cell_measurement_temperature ..... (K)        293 Check
PLAT200_ALERT_1_G Reported   _diffrn_ambient_temperature ..... (K)        293 Check
PLAT232_ALERT_2_G Hirshfeld Test Diff (M-X)  Cu1      --Cl1      .        7.2 s.u.
PLAT794_ALERT_5_G Tentative Bond Valency for Cu1       (II)      .       2.26 Info
PLAT899_ALERT_4_G SHELXL2018 is Deprecated and Succeeded by SHELXL     2019/3 Note
PLAT912_ALERT_4_G Missing # of FCF Reflections Above STh/L=  0.600        127 Note
PLAT941_ALERT_3_G Average HKL Measurement Multiplicity ..........         4.9 Low
PLAT978_ALERT_2_G Number C-C Bonds with Positive Residual Density.          4 Info
```

0 **ALERT level A** = Most likely a serious problem - resolve or explain
0 **ALERT level B** = A potentially serious problem, consider carefully
1 **ALERT level C** = Check. Ensure it is not caused by an omission or oversight
10 **ALERT level G** = General information/check it is not something unexpected

2 ALERT type 1 CIF construction/syntax error, inconsistent or missing data
2 ALERT type 2 Indicator that the structure model may be wrong or deficient
1 ALERT type 3 Indicator that the structure quality may be low
4 ALERT type 4 Improvement, methodology, query or suggestion
2 ALERT type 5 Informative message, check

It is advisable to attempt to resolve as many as possible of the alerts in all categories. Often the minor alerts point to easily fixed oversights, errors and omissions in your CIF or refinement strategy, so attention to these fine details can be worthwhile. In order to resolve some of the more serious problems it may be necessary to carry out additional measurements or structure refinements. However, the purpose of your study may justify the reported deviations and the more serious of these should normally be commented upon in the discussion or experimental section of a paper or in the "special_details" fields of the CIF. checkCIF was carefully designed to identify outliers and unusual parameters, but every test has its limitations and alerts that are not important in a particular case may appear. Conversely, the absence of alerts does not guarantee there are no aspects of the results needing attention. It is up to the individual to critically assess their own results and, if necessary, seek expert advice.

**Publication of your CIF in IUCr journals**

A basic structural check has been run on your CIF. These basic checks will be run on all CIFs submitted for publication in IUCr journals (*Acta Crystallographica*, *Journal of Applied Crystallography*, *Journal of Synchrotron Radiation*); however, if you intend to submit to *Acta Crystallographica Section C* or *E* or *IUCrData*, you should make sure that full publication checks are run on the final version of your CIF prior to submission.

**Publication of your CIF in other journals**

Please refer to the *Notes for Authors* of the relevant journal for any special instructions relating to CIF submission.

---

**PLATON version of 06/07/2023; check.def file version of 30/06/2023**

**Datablock ft602** - ellipsoid plot

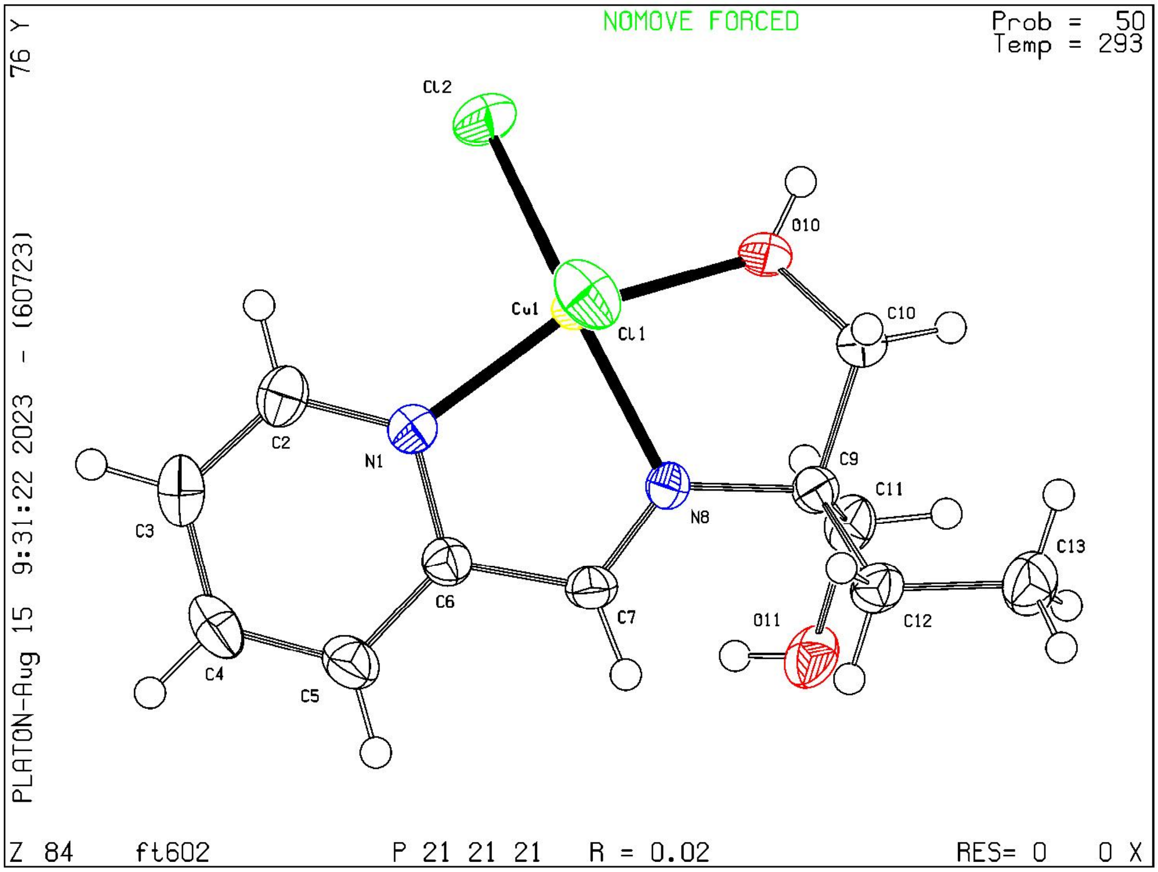

# checkCIF/PLATON report

Structure factors have been supplied for datablock(s) ft60-2-o3_a

THIS REPORT IS FOR GUIDANCE ONLY. IF USED AS PART OF A REVIEW PROCEDURE FOR PUBLICATION, IT SHOULD NOT REPLACE THE EXPERTISE OF AN EXPERIENCED CRYSTALLOGRAPHIC REFEREE.

No syntax errors found. CIF dictionary Interpreting this report

## Datablock: ft60-2-o3_a

---

| | | | |
|---|---|---|---|
| Bond precision: | C-C = 0.0079 A | | Wavelength=0.71073 |
| Cell: | a=9.6661(13) | b=11.621(2) | c=12.1882(17) |
| | alpha=90 | beta=90 | gamma=90 |
| Temperature: | 293 K | | |

| | Calculated | Reported |
|---|---|---|
| Volume | 1369.1(4) | 1369.0(4) |
| Space group | P 21 21 21 | P 21 21 21 |
| Hall group | P 2ac 2ab | P 2ac 2ab |
| Moiety formula | C11 H16 Cl2 Cu N2 O2 | C11 H16 Cl2 Cu N2 O2 |
| Sum formula | C11 H16 Cl2 Cu N2 O2 | C11 H16 Cl2 Cu N2 O2 |
| Mr | 342.71 | 342.70 |
| Dx,g cm-3 | 1.663 | 1.663 |
| Z | 4 | 4 |
| Mu (mm-1) | 1.980 | 1.980 |
| F000 | 700.0 | 700.0 |
| F000’ | 702.64 | |
| h,k,lmax | 12,15,16 | 12,15,15 |
| Nref | 3408[ 1953] | 2700 |
| Tmin,Tmax | 0.485,0.563 | 0.791,1.000 |
| Tmin’ | 0.476 | |

Correction method= # Reported T Limits: Tmin=0.791 Tmax=1.000
AbsCorr = MULTI-SCAN

Data completeness= 1.38/0.79 Theta(max)= 28.313

R(reflections)= 0.0404( 2398) wR2(reflections)= 0.0845( 2700)

S = 1.061 Npar= 163

The following ALERTS were generated. Each ALERT has the format
**test-name_ALERT_alert-type_alert-level.**
Click on the hyperlinks for more details of the test.

### Alert level C

```
PLAT341_ALERT_3_C Low Bond Precision on  C-C Bonds ...............    0.00789 Ang.
PLAT915_ALERT_3_C No Flack x Check Done: Low Friedel Pair Coverage         64 %
```

### Alert level G

```
PLAT007_ALERT_5_G Number of Unrefined Donor-H Atoms .............          2 Report
PLAT199_ALERT_1_G Reported _cell_measurement_temperature ..... (K)        293 Check
PLAT200_ALERT_1_G Reported   _diffrn_ambient_temperature ..... (K)        293 Check
PLAT480_ALERT_4_G Long H...A H-Bond Reported H2A      ..CL1      .       2.99 Ang.
PLAT794_ALERT_5_G Tentative Bond Valency for Cu1       (II)      .       2.26 Info
PLAT899_ALERT_4_G SHELXL2018 is Deprecated and Succeeded by SHELXL     2019/3 Note
PLAT912_ALERT_4_G Missing # of FCF Reflections Above STh/L=  0.600        171 Note
PLAT941_ALERT_3_G Average HKL Measurement Multiplicity ..........         2.0 Low
PLAT978_ALERT_2_G Number C-C Bonds with Positive Residual Density.          0 Info
```

0 **ALERT level A** = Most likely a serious problem - resolve or explain
0 **ALERT level B** = A potentially serious problem, consider carefully
2 **ALERT level C** = Check. Ensure it is not caused by an omission or oversight
9 **ALERT level G** = General information/check it is not something unexpected

2 ALERT type 1 CIF construction/syntax error, inconsistent or missing data
1 ALERT type 2 Indicator that the structure model may be wrong or deficient
3 ALERT type 3 Indicator that the structure quality may be low
3 ALERT type 4 Improvement, methodology, query or suggestion
2 ALERT type 5 Informative message, check

It is advisable to attempt to resolve as many as possible of the alerts in all categories. Often the minor alerts point to easily fixed oversights, errors and omissions in your CIF or refinement strategy, so attention to these fine details can be worthwhile. In order to resolve some of the more serious problems it may be necessary to carry out additional measurements or structure refinements. However, the purpose of your study may justify the reported deviations and the more serious of these should normally be commented upon in the discussion or experimental section of a paper or in the "special_details" fields of the CIF. checkCIF was carefully designed to identify outliers and unusual parameters, but every test has its limitations and alerts that are not important in a particular case may appear. Conversely, the absence of alerts does not guarantee there are no aspects of the results needing attention. It is up to the individual to critically assess their own results and, if necessary, seek expert advice.

**Publication of your CIF in IUCr journals**

A basic structural check has been run on your CIF. These basic checks will be run on all CIFs submitted for publication in IUCr journals (*Acta Crystallographica*, *Journal of Applied Crystallography*, *Journal of Synchrotron Radiation*); however, if you intend to submit to *Acta Crystallographica Section C* or *E* or *IUCrData*, you should make sure that full publication checks are run on the final version of your CIF prior to submission.

**Publication of your CIF in other journals**

Please refer to the *Notes for Authors* of the relevant journal for any special instructions relating to CIF submission.

---

**PLATON version of 06/07/2023; check.def file version of 30/06/2023**

**Datablock ft60-2-o3_a** - ellipsoid plot

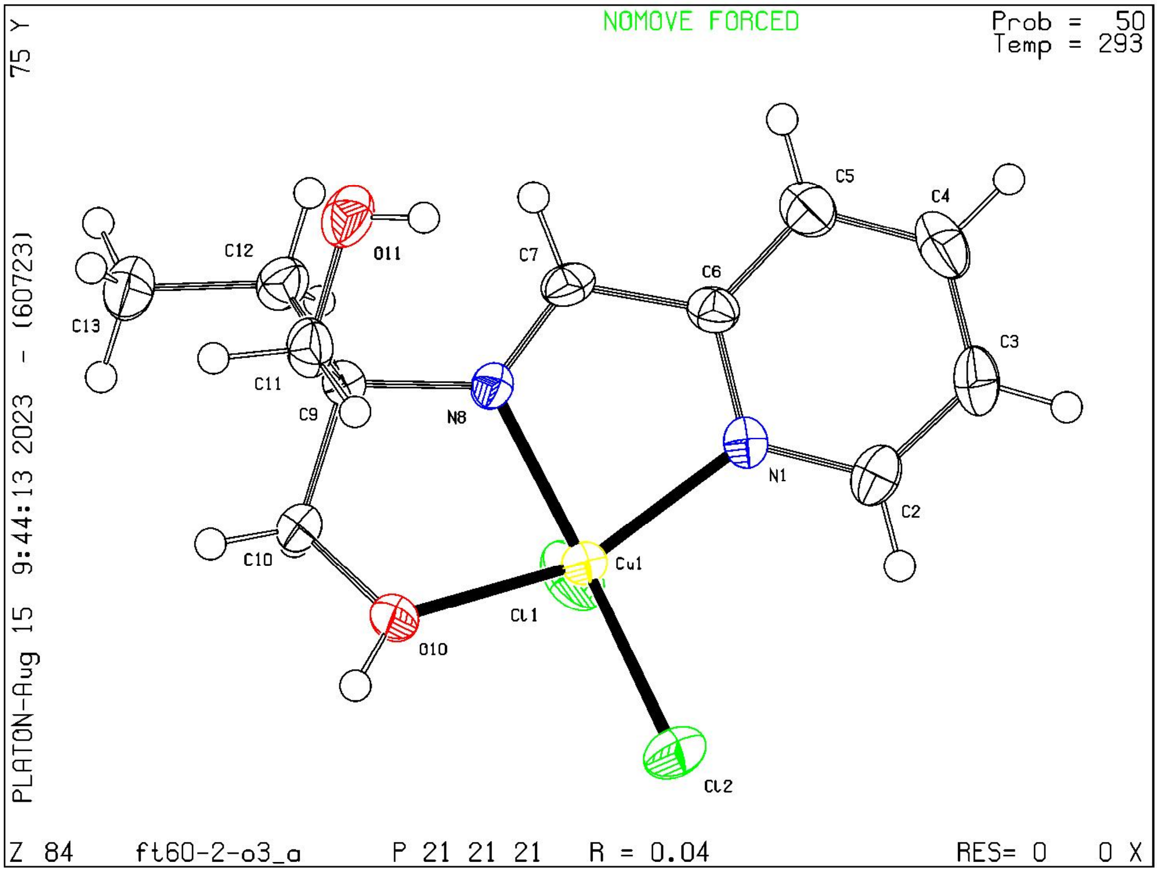

# checkCIF/PLATON report

Structure factors have been supplied for datablock(s) ft182-2b-g_a

THIS REPORT IS FOR GUIDANCE ONLY. IF USED AS PART OF A REVIEW PROCEDURE FOR PUBLICATION, IT SHOULD NOT REPLACE THE EXPERTISE OF AN EXPERIENCED CRYSTALLOGRAPHIC REFEREE.

No syntax errors found. CIF dictionary Interpreting this report

## Datablock: ft182-2b-g_a

---

```
Bond precision:   C-C = 0.0086 A          Wavelength=1.54184

Cell:              a=9.7775(7)     b=11.7383(9)     c=12.3077(10)
                   alpha=90        beta=90          gamma=90
Temperature:       100 K
```

| | Calculated | Reported |
|---|---|---|
| Volume | 1412.57(19) | 1412.57(19) |
| Space group | P 21 21 21 | P 21 21 21 |
| Hall group | P 2ac 2ab | P 2ac 2ab |
| Moiety formula | C11 H16 Br2 Cu N2 O2 | C11 H16 Br2 Cu N2 O2 |
| Sum formula | C11 H16 Br2 Cu N2 O2 | C11 H16 Br2 Cu N2 O2 |
| Mr | 431.61 | 431.62 |
| Dx,g cm-3 | 2.030 | 2.030 |
| Z | 4 | 4 |
| Mu (mm-1) | 8.785 | 8.785 |
| F000 | 844.0 | 844.0 |
| F000' | 832.08 | |
| h,k,lmax | 12,14,15 | 11,14,15 |
| Nref | 3016[ 1737] | 2528 |
| Tmin,Tmax | 0.220,0.287 | 0.809,1.000 |
| Tmin' | 0.140 | |

```
Correction method= # Reported T Limits: Tmin=0.809 Tmax=1.000
AbsCorr = MULTI-SCAN

Data completeness= 1.46/0.84       Theta(max)= 77.993

R(reflections)= 0.0304( 2515)              wR2(reflections)=
                                           0.0791( 2528)
S = 1.067                  Npar= 164
```

The following ALERTS were generated. Each ALERT has the format

**test-name_ALERT_alert-type_alert-level.**

Click on the hyperlinks for more details of the test.

## Alert level B

```
PLAT417_ALERT_2_B Short Inter D-H..H-D       H10C     ..H11C     .       2.07 Ang.
                                         3/2-x,1-y,-1/2+z  =      2_664 Check
```

## Alert level C

```
PLAT341_ALERT_3_C Low Bond Precision on  C-C Bonds ...............    0.00856 Ang.
PLAT911_ALERT_3_C Missing FCF Refl Between Thmin & STh/L=    0.600          3 Report
PLAT915_ALERT_3_C No Flack x Check Done: Low Friedel Pair Coverage         71 %
```

## Alert level G

```
PLAT002_ALERT_2_G Number of Distance or Angle Restraints on AtSite          2 Note
PLAT007_ALERT_5_G Number of Unrefined Donor-H Atoms ..............          2 Report
PLAT172_ALERT_4_G The CIF-Embedded .res File Contains DFIX Records          1 Report
PLAT232_ALERT_2_G Hirshfeld Test Diff (M-X)  Br1      --Cu1      .        5.6 s.u.
PLAT480_ALERT_4_G Long H...A H-Bond Reported H2A      ..BR2      .       3.03 Ang.
PLAT480_ALERT_4_G Long H...A H-Bond Reported H2A      ..BR1      .       3.04 Ang.
PLAT480_ALERT_4_G Long H...A H-Bond Reported H7A      ..BR2      .       3.05 Ang.
PLAT480_ALERT_4_G Long H...A H-Bond Reported H10A     ..BR1      .       3.12 Ang.
PLAT480_ALERT_4_G Long H...A H-Bond Reported H11B     ..BR2      .       3.10 Ang.
PLAT794_ALERT_5_G Tentative Bond Valency for Cu1       (II)      .       1.98 Info
PLAT860_ALERT_3_G Number of Least-Squares Restraints .............          1 Note
PLAT899_ALERT_4_G SHELXL2018 is Deprecated and Succeeded by SHELXL     2019/3 Note
PLAT912_ALERT_4_G Missing # of FCF Reflections Above STh/L=  0.600         97 Note
PLAT941_ALERT_3_G Average HKL Measurement Multiplicity ...........        3.7 Low
PLAT961_ALERT_5_G Dataset Contains no Negative Intensities .......     Please Check
PLAT978_ALERT_2_G Number C-C Bonds with Positive Residual Density.          0 Info
```

0 **ALERT level A** = Most likely a serious problem - resolve or explain
1 **ALERT level B** = A potentially serious problem, consider carefully
3 **ALERT level C** = Check. Ensure it is not caused by an omission or oversight
16 **ALERT level G** = General information/check it is not something unexpected

0 ALERT type 1 CIF construction/syntax error, inconsistent or missing data
4 ALERT type 2 Indicator that the structure model may be wrong or deficient
5 ALERT type 3 Indicator that the structure quality may be low
8 ALERT type 4 Improvement, methodology, query or suggestion
3 ALERT type 5 Informative message, check

It is advisable to attempt to resolve as many as possible of the alerts in all categories. Often the minor alerts point to easily fixed oversights, errors and omissions in your CIF or refinement strategy, so attention to these fine details can be worthwhile. In order to resolve some of the more serious problems it may be necessary to carry out additional measurements or structure refinements. However, the purpose of your study may justify the reported deviations and the more serious of these should normally be commented upon in the discussion or experimental section of a paper or in the "special_details" fields of the CIF. checkCIF was carefully designed to identify outliers and unusual parameters, but every test has its limitations and alerts that are not important in a particular case may appear. Conversely, the absence of alerts does not guarantee there are no aspects of the results needing attention. It is up to the individual to critically assess their own results and, if necessary, seek expert advice.

**Publication of your CIF in IUCr journals**

A basic structural check has been run on your CIF. These basic checks will be run on all CIFs submitted for publication in IUCr journals (*Acta Crystallographica*, *Journal of Applied Crystallography*, *Journal of Synchrotron Radiation*); however, if you intend to submit to *Acta Crystallographica Section C* or *E* or *IUCrData*, you should make sure that full publication checks are run on the final version of your CIF prior to submission.

**Publication of your CIF in other journals**

Please refer to the *Notes for Authors* of the relevant journal for any special instructions relating to CIF submission.

---

**PLATON version of 06/07/2023; check.def file version of 30/06/2023**

**Datablock ft182-2b-g_a** - ellipsoid plot

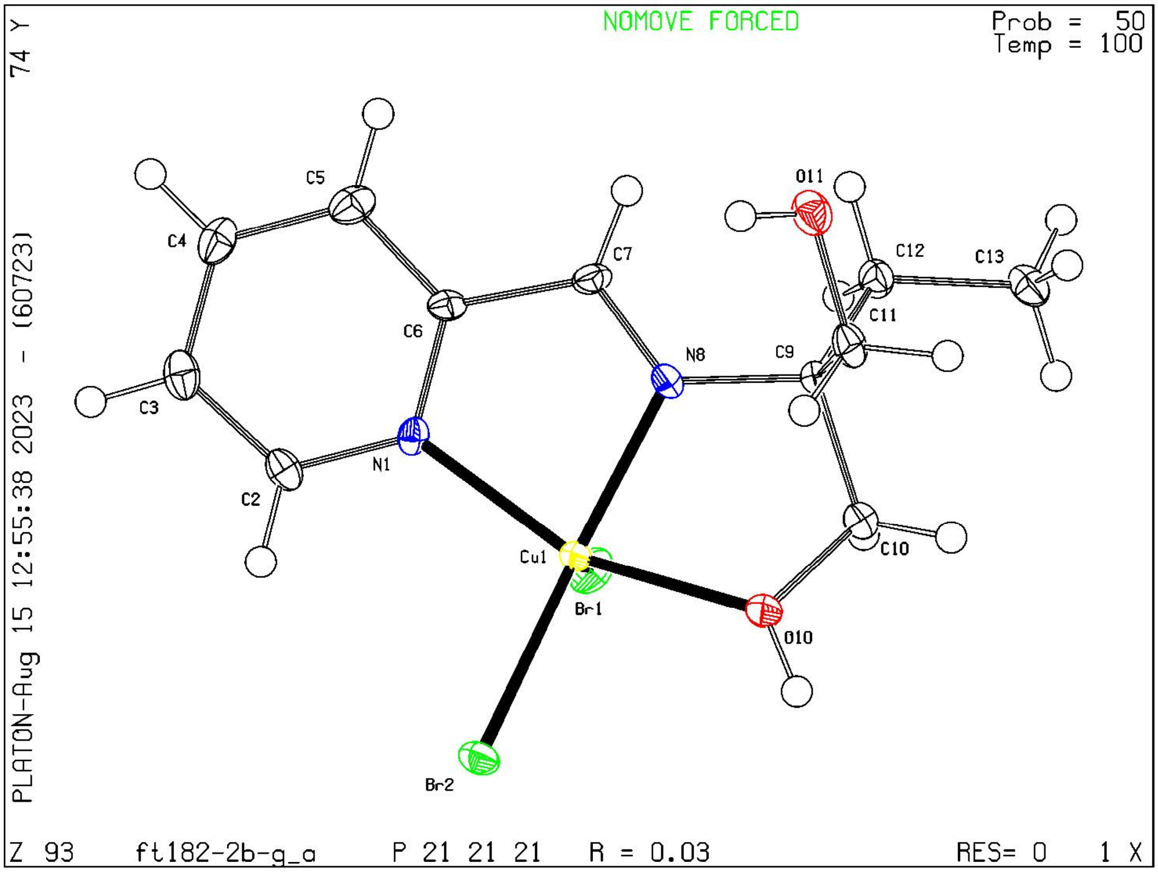

# checkCIF/PLATON report

Structure factors have been supplied for datablock(s) m1a

THIS REPORT IS FOR GUIDANCE ONLY. IF USED AS PART OF A REVIEW PROCEDURE FOR PUBLICATION, IT SHOULD NOT REPLACE THE EXPERTISE OF AN EXPERIENCED CRYSTALLOGRAPHIC REFEREE.

No syntax errors found. CIF dictionary Interpreting this report

## Datablock: m1a

---

| | | | |
|---|---|---|---|
| Bond precision: | C-C = 0.0162 A | | Wavelength=0.71073 |
| Cell: | a=5.6181(12) | b=16.267(3) | c=16.890(3) |
| | alpha=90 | beta=96.578(19) | gamma=90 |
| Temperature: | 293 K | | |

| | Calculated | Reported |
|---|---|---|
| Volume | 1533.4(5) | 1533.5(5) |
| Space group | P 21 | P 21 |
| Hall group | P 2yb | P 2yb |
| Moiety formula | C17 H17 N3 O2 | C17 H17 N3 O2 |
| Sum formula | C17 H17 N3 O2 | C17 H17 N3 O2 |
| Mr | 295.34 | 295.33 |
| Dx,g cm-3 | 1.279 | 1.279 |
| Z | 4 | 4 |
| Mu (mm-1) | 0.086 | 0.086 |
| F000 | 624.0 | 624.0 |
| F000’ | 624.26 | |
| h,k,lmax | 7,21,22 | 7,21,22 |
| Nref | 7784[ 4025] | 5896 |
| Tmin,Tmax | 0.987,0.996 | 0.965,0.985 |
| Tmin’ | 0.957 | |

Correction method= # Reported T Limits: Tmin=0.965 Tmax=0.985
AbsCorr = ANALYTICAL

Data completeness= 1.46/0.76 Theta(max)= 28.500

R(reflections)= 0.1183( 2251) wR2(reflections)= 0.1648( 5896)

S = 0.999 Npar= 407

The following ALERTS were generated. Each ALERT has the format
**test-name_ALERT_alert-type_alert-level.**
Click on the hyperlinks for more details of the test.

### Alert level B

```
PLAT026_ALERT_3_B Ratio Observed / Unique Reflections (too) Low ..         38% Check
PLAT340_ALERT_3_B Low Bond Precision on  C-C Bonds ...............    0.01622 Ang.
```

### Alert level C

```
STRVA01_ALERT_2_C           Chirality of atom sites is inverted?
           From the CIF: _refine_ls_abs_structure_Flack    0.800
           From the CIF: _refine_ls_abs_structure_Flack_su    1.000
PLAT082_ALERT_2_C High R1 Value ..................................       0.12 Report
PLAT234_ALERT_4_C Large Hirshfeld Difference C20A     --C21A     .       0.17 Ang.
PLAT241_ALERT_2_C High   'MainMol' Ueq as Compared to Neighbors of       C12B Check
PLAT242_ALERT_2_C Low    'MainMol' Ueq as Compared to Neighbors of       C17B Check
PLAT906_ALERT_3_C Large K Value in the Analysis of Variance ......     13.854 Check
PLAT906_ALERT_3_C Large K Value in the Analysis of Variance ......      3.097 Check
PLAT906_ALERT_3_C Large K Value in the Analysis of Variance ......      6.403 Check
PLAT906_ALERT_3_C Large K Value in the Analysis of Variance ......      3.374 Check
PLAT906_ALERT_3_C Large K Value in the Analysis of Variance ......      2.938 Check
PLAT906_ALERT_3_C Large K Value in the Analysis of Variance ......      2.473 Check
PLAT907_ALERT_2_C Flack x > 0.5, Structure Needs to be Inverted? .       0.80 Check
PLAT911_ALERT_3_C Missing FCF Refl Between Thmin & STh/L=    0.600          5 Report
PLAT915_ALERT_3_C No Flack x Check Done: Low Friedel Pair Coverage         61 %
```

### Alert level G

```
PLAT002_ALERT_2_G Number of Distance or Angle Restraints on AtSite          4 Note
PLAT007_ALERT_5_G Number of Unrefined Donor-H Atoms ..............          3 Report
PLAT032_ALERT_4_G Std. Uncertainty on Flack Parameter Value High .      1.000 Report
PLAT172_ALERT_4_G The CIF-Embedded .res File Contains DFIX Records          1 Report
PLAT199_ALERT_1_G Reported _cell_measurement_temperature ..... (K)        293 Check
PLAT200_ALERT_1_G Reported   _diffrn_ambient_temperature ..... (K)        293 Check
PLAT300_ALERT_4_G Atom Site Occupancy of O14A       Constrained at        0.8 Check
PLAT300_ALERT_4_G Atom Site Occupancy of O14C       Constrained at        0.2 Check
PLAT300_ALERT_4_G Atom Site Occupancy of H13A       Constrained at        0.8 Check
PLAT300_ALERT_4_G Atom Site Occupancy of H14A       Constrained at        0.8 Check
PLAT300_ALERT_4_G Atom Site Occupancy of H13C       Constrained at        0.2 Check
PLAT300_ALERT_4_G Atom Site Occupancy of H14C       Constrained at        0.2 Check
PLAT301_ALERT_3_G Main Residue  Disorder ..............(Resd  1  )         5% Note
PLAT415_ALERT_2_G Short Inter D-H..H-X       H13B      ..H14A     .       2.13 Ang.
                                                   x,y,z  =      1_555 Check
PLAT417_ALERT_2_G Short Inter D-H..H-D       H14A      ..H14B     .       1.91 Ang.
                                                   x,y,z  =      1_555 Check
PLAT480_ALERT_4_G Long H...A H-Bond Reported H13A      ..N10A     .       2.70 Ang.
PLAT480_ALERT_4_G Long H...A H-Bond Reported H15A      ..O14C     .       2.63 Ang.
PLAT720_ALERT_4_G Number of Unusual/Non-Standard Labels ..........         10 Note
PLAT728_ALERT_1_G D-H..A  Calc      139.00, Rep      137.80 Dev...       1.20 Degree
              O14C    -H13A    -O14A       1_555  1_555  1_555     #    3 Check
PLAT779_ALERT_4_G Suspect or Irrelevant (Bond) Angle(s) in CIF ...       1.00 Deg.
              O14C -C13A -H13A    1_555    1_555    1_555 ......     #   43 Check
PLAT779_ALERT_4_G Suspect or Irrelevant (Bond) Angle(s) in CIF ...       0.80 Deg.
```

```
              O14A -C13A -H13C    1_555   1_555   1_555 ......     #   46 Check
PLAT779_ALERT_4_G Suspect or Irrelevant (Bond) Angle(s) in CIF ...       1.70 Deg.
              C13A -O14A -H13C    1_555   1_555   1_555 ......     #   51 Check
PLAT779_ALERT_4_G Suspect or Irrelevant (Bond) Angle(s) in CIF ...       1.90 Deg.
              C13A -O14C -H13A    1_555   1_555   1_555 ......     #   54 Check
PLAT860_ALERT_3_G Number of Least-Squares Restraints ............          3 Note
PLAT899_ALERT_4_G SHELXL2018 is Deprecated and Succeeded by SHELXL     2019/3 Note
PLAT910_ALERT_3_G Missing # of FCF Reflection(s) Below Theta(Min).         2 Note
PLAT912_ALERT_4_G Missing # of FCF Reflections Above STh/L=  0.600       407 Note
PLAT916_ALERT_2_G Hooft y and Flack x Parameter Values Differ by .      0.10 Check
PLAT933_ALERT_2_G Number of HKL-OMIT Records in Embedded .res File         1 Note
PLAT941_ALERT_3_G Average HKL Measurement Multiplicity ..........        2.8 Low
PLAT965_ALERT_2_G The SHELXL WEIGHT Optimisation has not Converged     Please Check
PLAT978_ALERT_2_G Number C-C Bonds with Positive Residual Density.         0 Info
```

---

0 **ALERT level A** = Most likely a serious problem - resolve or explain
2 **ALERT level B** = A potentially serious problem, consider carefully
14 **ALERT level C** = Check. Ensure it is not caused by an omission or oversight
32 **ALERT level G** = General information/check it is not something unexpected

3 ALERT type 1 CIF construction/syntax error, inconsistent or missing data
12 ALERT type 2 Indicator that the structure model may be wrong or deficient
14 ALERT type 3 Indicator that the structure quality may be low
18 ALERT type 4 Improvement, methodology, query or suggestion
1 ALERT type 5 Informative message, check

---

It is advisable to attempt to resolve as many as possible of the alerts in all categories. Often the minor alerts point to easily fixed oversights, errors and omissions in your CIF or refinement strategy, so attention to these fine details can be worthwhile. In order to resolve some of the more serious problems it may be necessary to carry out additional measurements or structure refinements. However, the purpose of your study may justify the reported deviations and the more serious of these should normally be commented upon in the discussion or experimental section of a paper or in the "special_details" fields of the CIF. checkCIF was carefully designed to identify outliers and unusual parameters, but every test has its limitations and alerts that are not important in a particular case may appear. Conversely, the absence of alerts does not guarantee there are no aspects of the results needing attention. It is up to the individual to critically assess their own results and, if necessary, seek expert advice.

**Publication of your CIF in IUCr journals**

A basic structural check has been run on your CIF. These basic checks will be run on all CIFs submitted for publication in IUCr journals (*Acta Crystallographica*, *Journal of Applied Crystallography*, *Journal of Synchrotron Radiation*); however, if you intend to submit to *Acta Crystallographica Section C* or *E* or *IUCrData*, you should make sure that full publication checks are run on the final version of your CIF prior to submission.

**Publication of your CIF in other journals**

Please refer to the *Notes for Authors* of the relevant journal for any special instructions relating to CIF submission.

---

**PLATON version of 06/07/2023; check.def file version of 30/06/2023**

**Datablock m1a** - ellipsoid plot

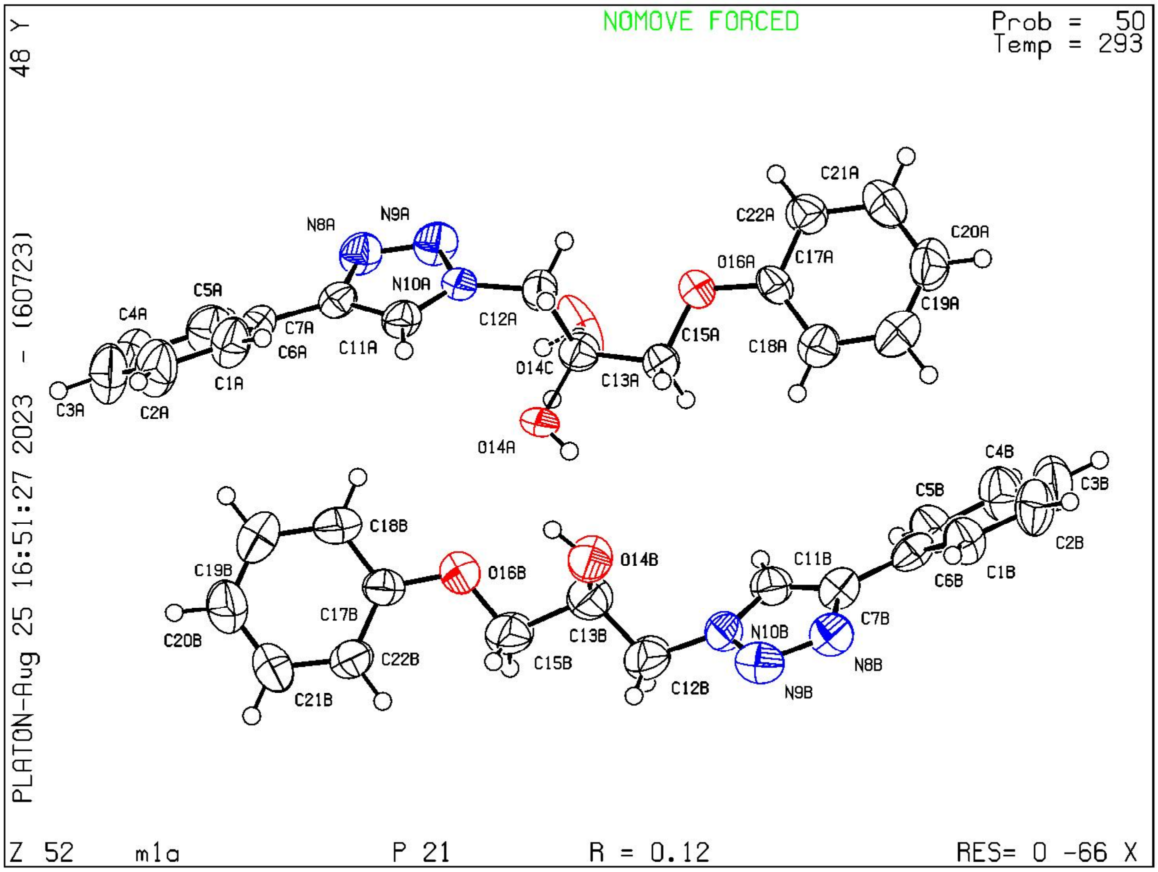

Supplement: Supplementary file 1 — Supplementary Information 1. [file 41598_2024_60930_MOESM1_ESM.pdf]
