# Supplementary material for: Synthesis of chiral Cu(II) complexes from pro-chiral Schiff base ligand and investigation of their catalytic activity in the asymmetric synthesis of 1,2,3-triazoles
Source: Sci Rep. 2024 May 8;14:10603. doi: 10.1038/s41598-024-60930-w (PMC11079015; doi:10.1038/s41598-024-60930-w)
Supplement: Supplementary file 6 — Supplementary Information 6. [file 41598_2024_60930_MOESM6_ESM.doc]

**Synthesis of chiral Cu(II) complexes from pro-chiral Schiff base ligand and investigation of their catalytic activity in the asymmetric synthesis of 1,2,3-triazoles**

Fatemeh Ajormal,^a^ Rahman Bikas,^b,*^ Nader Noshiranzadeh,^a,*^ Marzieh Emami,^a^ Anna Kozakiewicz-Piekarz,^c^

*^a^ Department of Chemistry, Faculty of Science, University of Zanjan, 45371-38791, Zanjan, Iran*

*^b^ Department of Chemistry, Faculty of Science, Imam Khomeini International University,34148-96818, Qazvin, Iran*

*^c^ Department of Biomedical and Polymer Chemistry, Faculty of Chemistry, Nicolaus Copernicus University in Torun, 87-100, Torun, Poland*

* Corresponding authors; Email addresses: [bikas@sci.ikiu.ac.ir](mailto:bikas@sci.ikiu.ac.ir), (R. Bikas), [nadernoshiranzadeh@yahoo.com](mailto:nadernoshiranzadeh@yahoo.com) (N. Noshiranzadeh)

***Materials and instrumentation***

All used materials including copper(II) bromide, copper(II) chloride dihydrate, 2-amino-2-ethyl-1,3-propanediol, pyridine-2-carbaldehyde, sodium azide, and phenylacetylene and the solvents with highest grade are commercially available and were ordered from the Sigma Aldrich or Alfa Aesar companies. These compounds were used without further purification process. Fourier transform infrared spectroscopy (FT-IR) was used for identifying reaction compounds by applying a Bruker TENSOR 27 FT–IR spectrometer. ^1^H NMR and ^13^C NMR spectra were recorded on a Bruker Avance (250 and 62.9 MHz) spectrometer in CDCl_3_. UV–Vis spectra of the samples were measured with a thermo-spectronic Helios Alpha spectrophotometer. The spectroscopic measurements were recorded under ambient conditions using methanol solvent in the range of 200–800 nm. CD spectra were measured with a circular dichroism spectrometer model-215 (USA). Atomic absorption analyses were acquired using Varian Spectra AA-220 equipment.

***Single crystal X-ray analysis***

The diffraction data of S- and R-enantiomer of (**1**) and **T1** were collected at 293(2) K on an Oxford Sapphire CCD diffractometer using MoKα radiation (λ = 0.71073 Å), while crystal of (**2**) was measured at 100(2) K on Rigaku XtaLAB Synergy-S (Dualflex) diffractometer with monochromatic CuKα X-ray source (λ = 1.54184 Å). The data were processed using *CrysAlisPro*.[[1]](#endnote-2) All structures were solved by direct methods and refined with the full-matrix least-squares procedure on *F^2^* (SHELX2014 and SHELX2018).[[2]](#endnote-3) Positions of hydrogen atoms attached to oxygen atoms were found in the electron density maps, whereas hydrogen atoms onto carbon atoms were positioned with the idealized geometry. The structural data have been deposited at the Cambridge Crystallographic Data Centre (www.ccdc.cam.ac.uk/data_request/cif). CCDC 2288813-2288815 and 1921722 contain crystallographic data for S-(**1**), R-(**1**), R-(**2**), and **T1**, respectively.


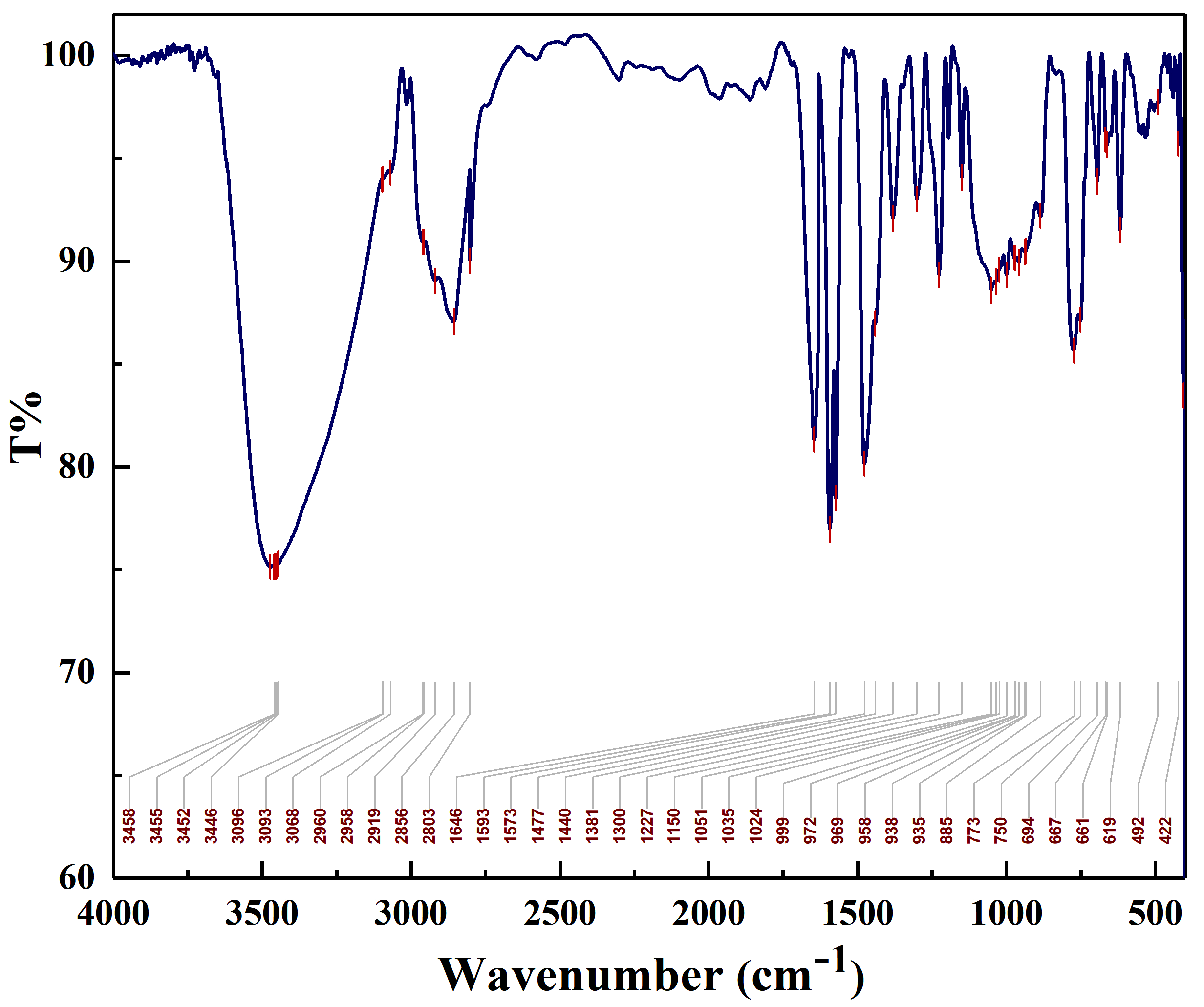


Fig. S1: The FT-IR spectrum of the ligand HL on KBr disk


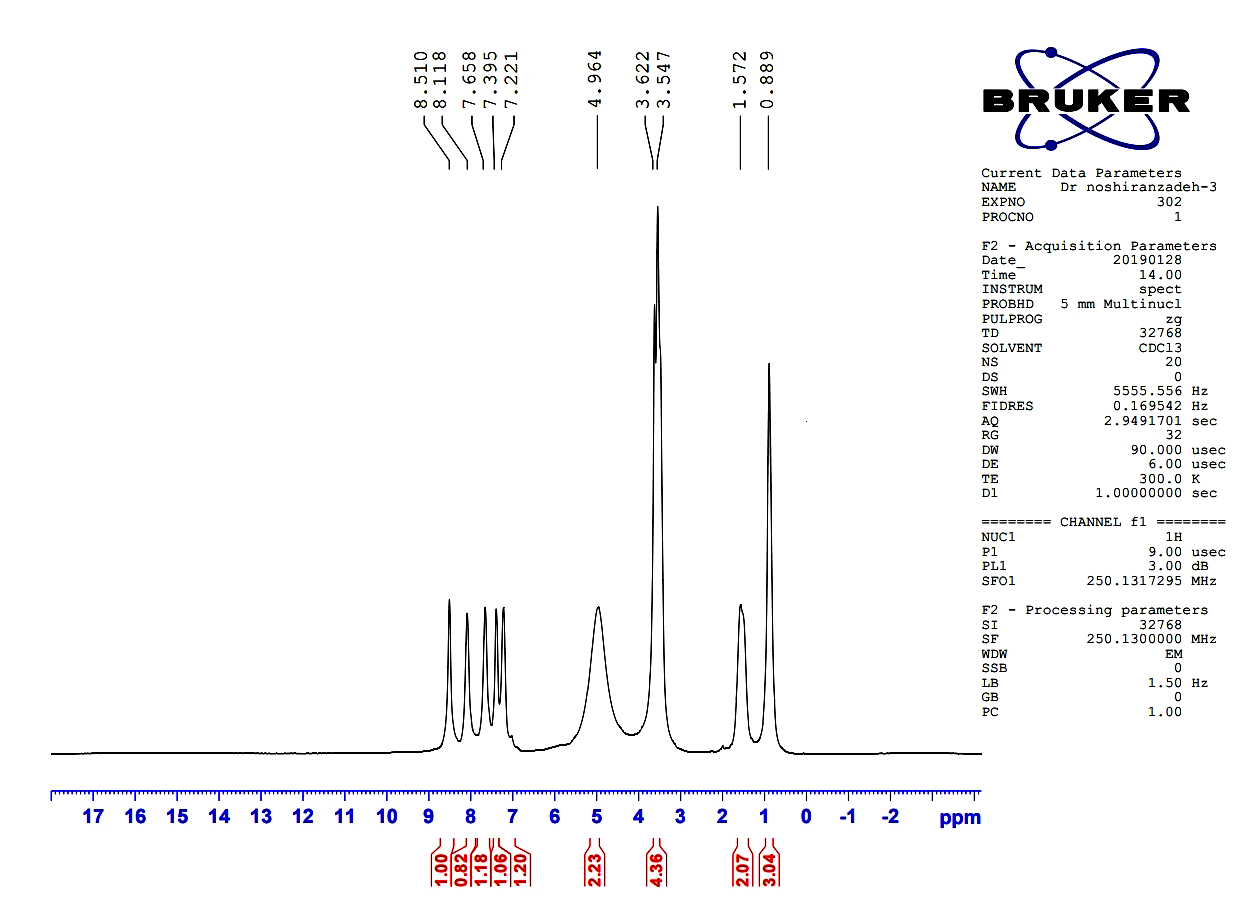


Fig. S2: ^1^H NMR spectrum of the ligand HL in CDCl_3_


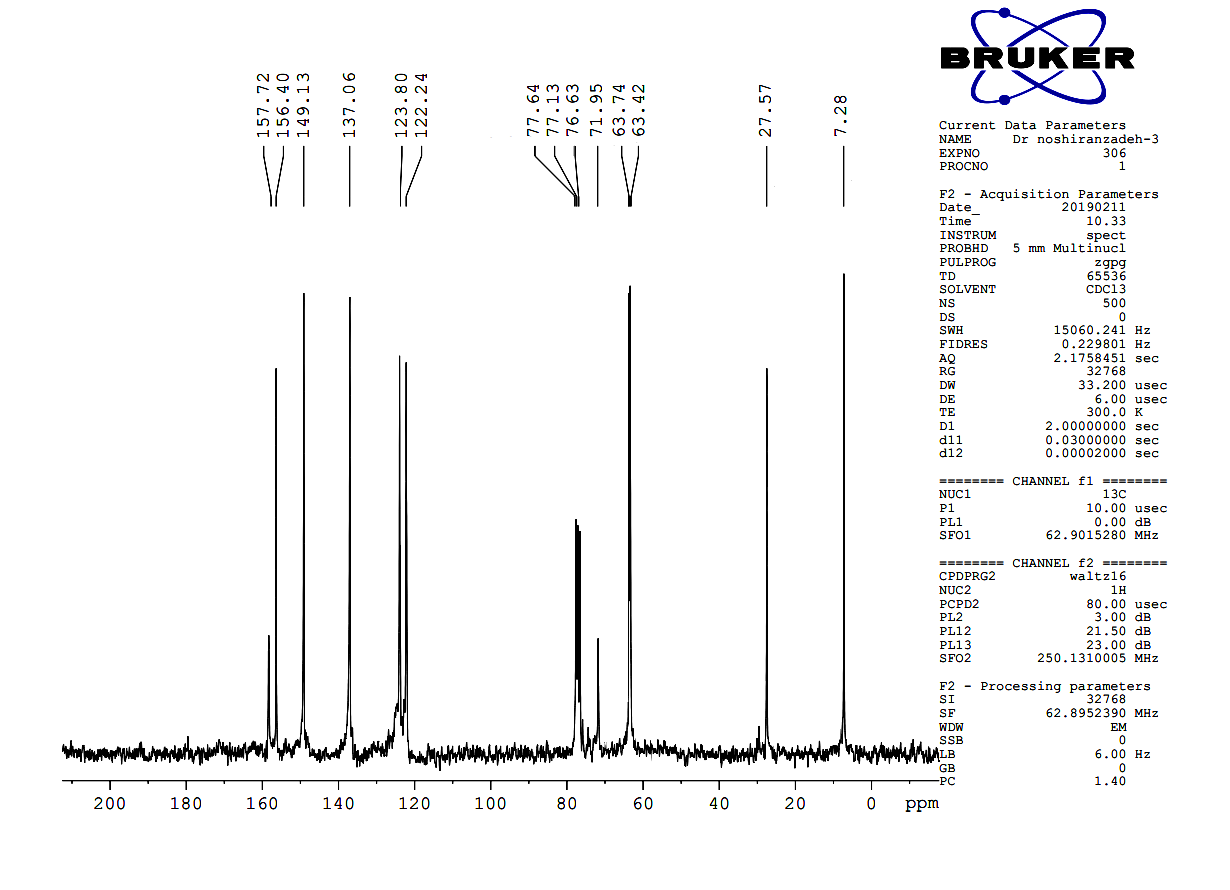


Fig. S3: ^13^ C NMR spectrum of the ligand HL in CDCl_3_


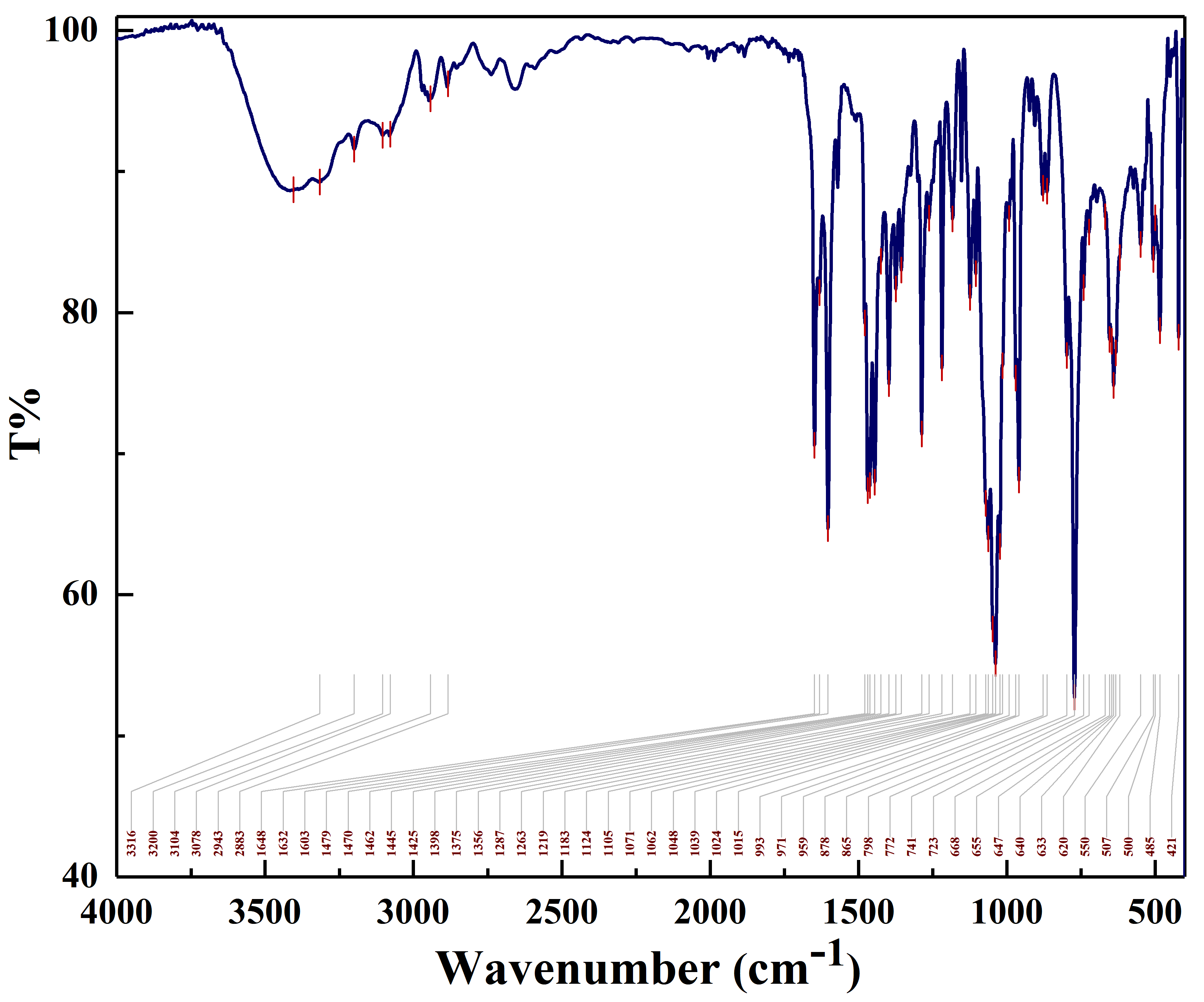


Fig. S4: The FT-IR spectrum of complex [Cu(HL)Cl_2_] on KBr disk


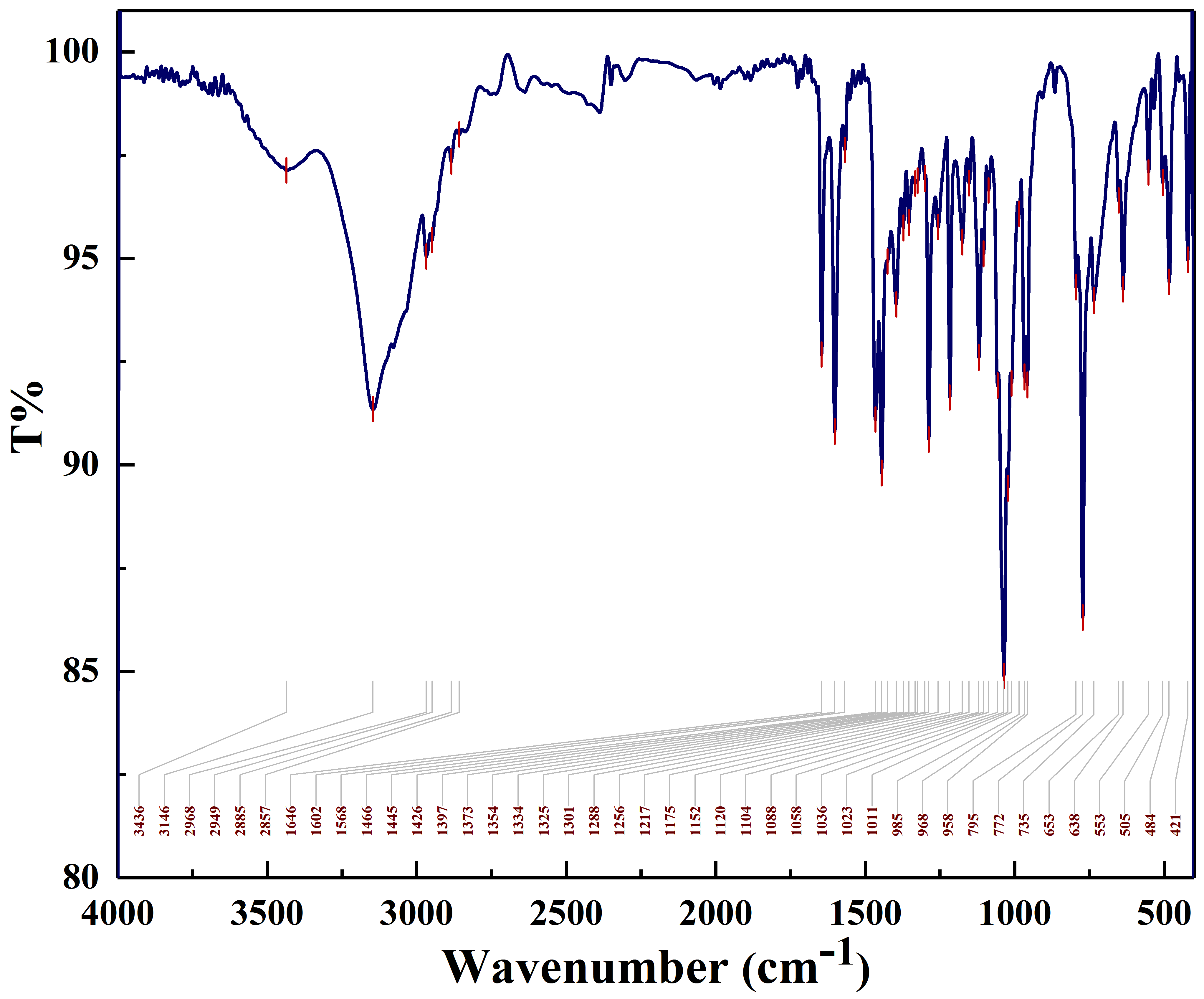


Fig. S5: The FT-IR spectrum of complex [Cu(HL)Br_2_] on KBr disk


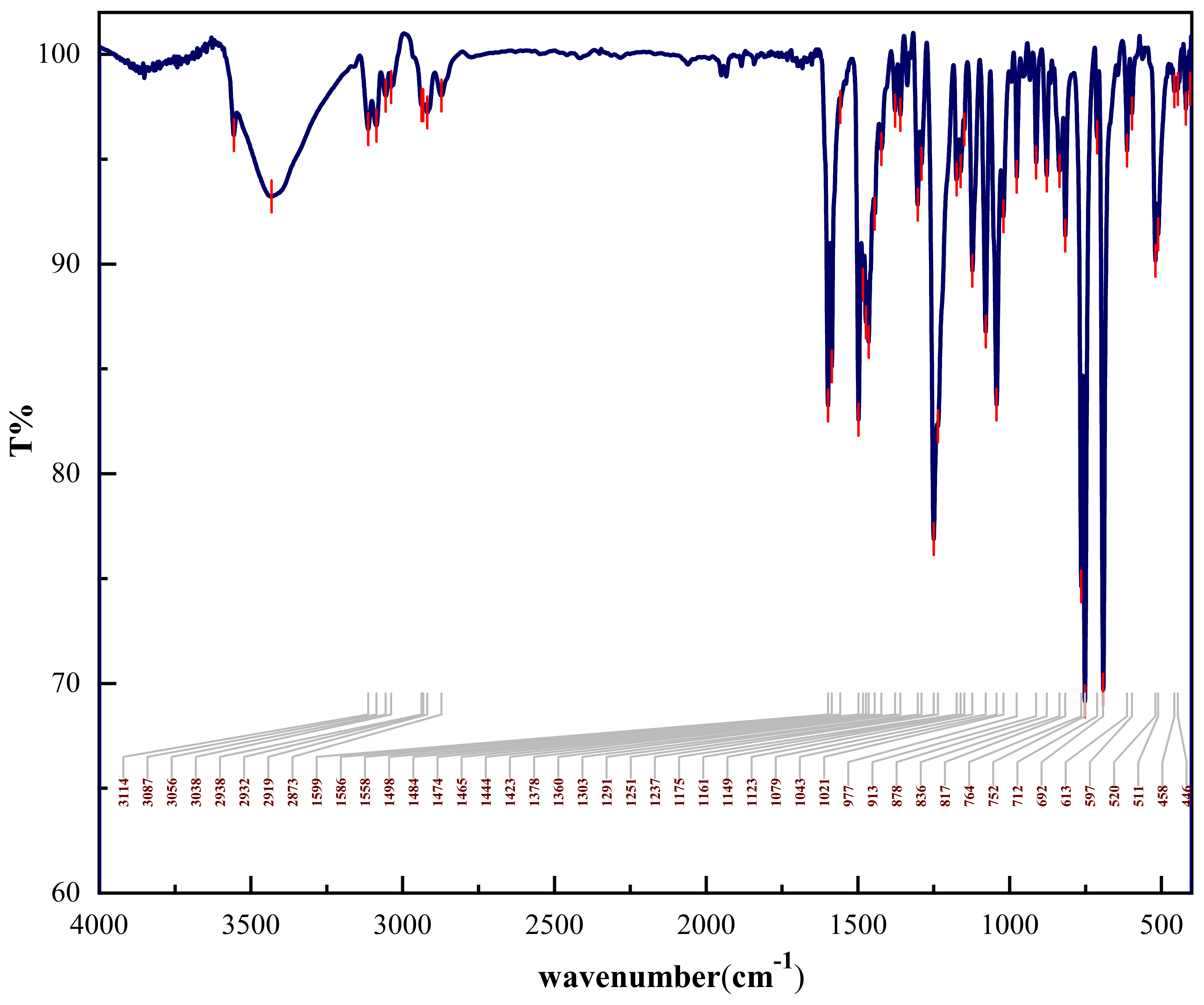


Fig. S6: The FT-IR spectrum of synthesized **T1** on KBr disk


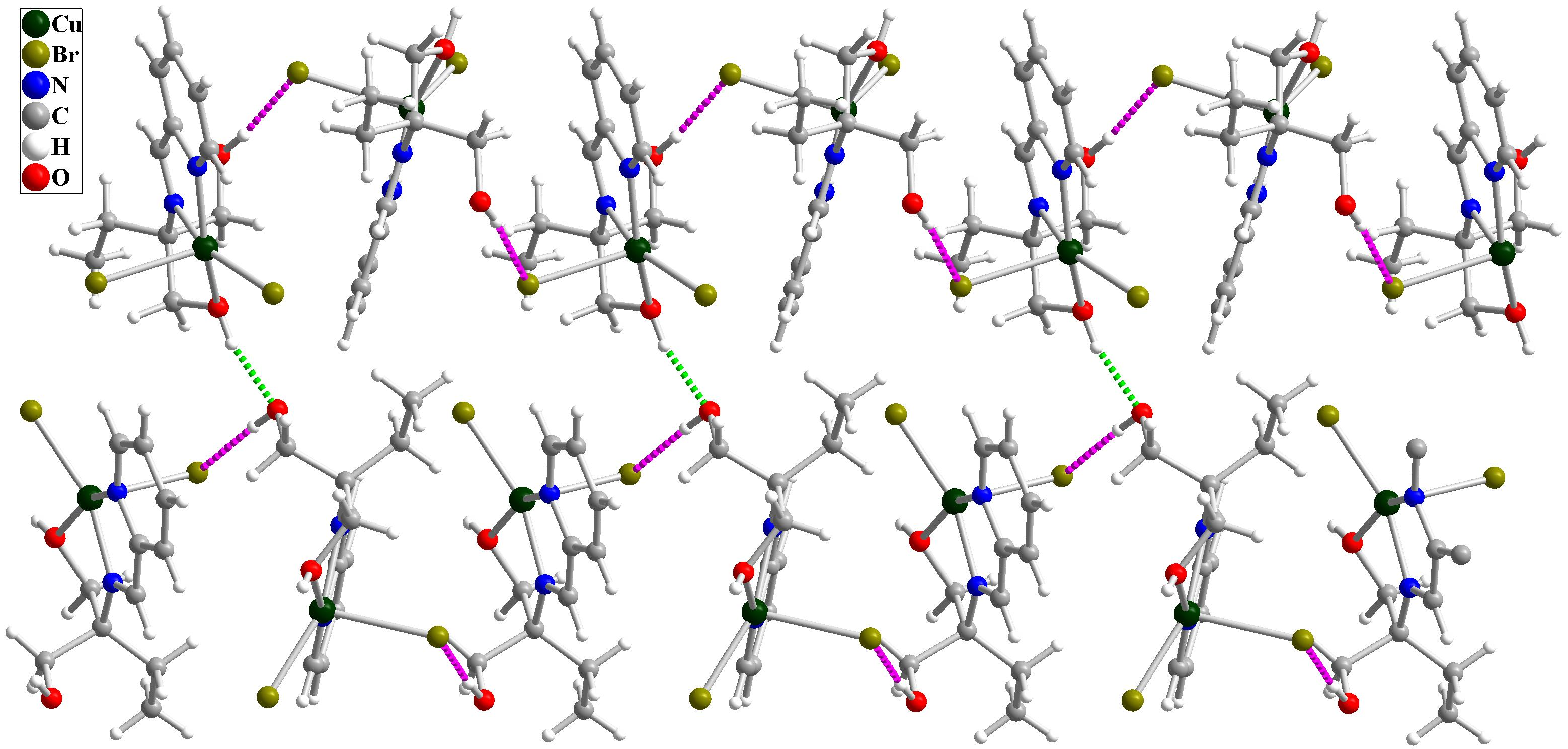


Fig. S7: Part of intermolecular hydrogen bond interactions in the crystal structure of [Cu(HL)Br_2_] which are shown as pink and green dashed lines


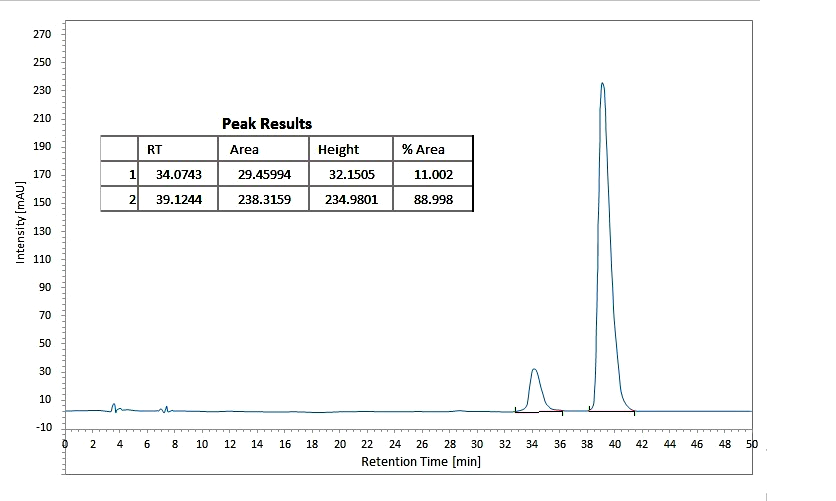


Fig. S8: Chiral analyses obtained by HPLC chromatograms of the separated R-[Cu(HL) Cl_2_] enantiomer, Conditions: 4.6 × 250 mm CHIRALPAK IC column, methanol /acetonitrile 90:10; flow rate of 0.5 mL/min, and the column temperature was set at 25 °C.


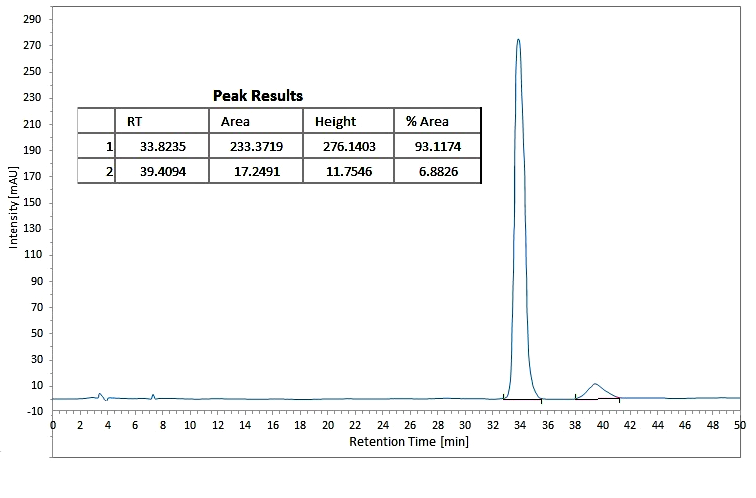


Fig. S9: Chiral analyses obtained by HPLC chromatograms of the separated S-[Cu(HL) Cl_2_] enantiomer, Conditions: 4.6 × 250 mm CHIRALPAK IC column, methanol /acetonitrile 90:10; flow rate of 0.5 mL/min, and the column temperature was set at 25 °C.


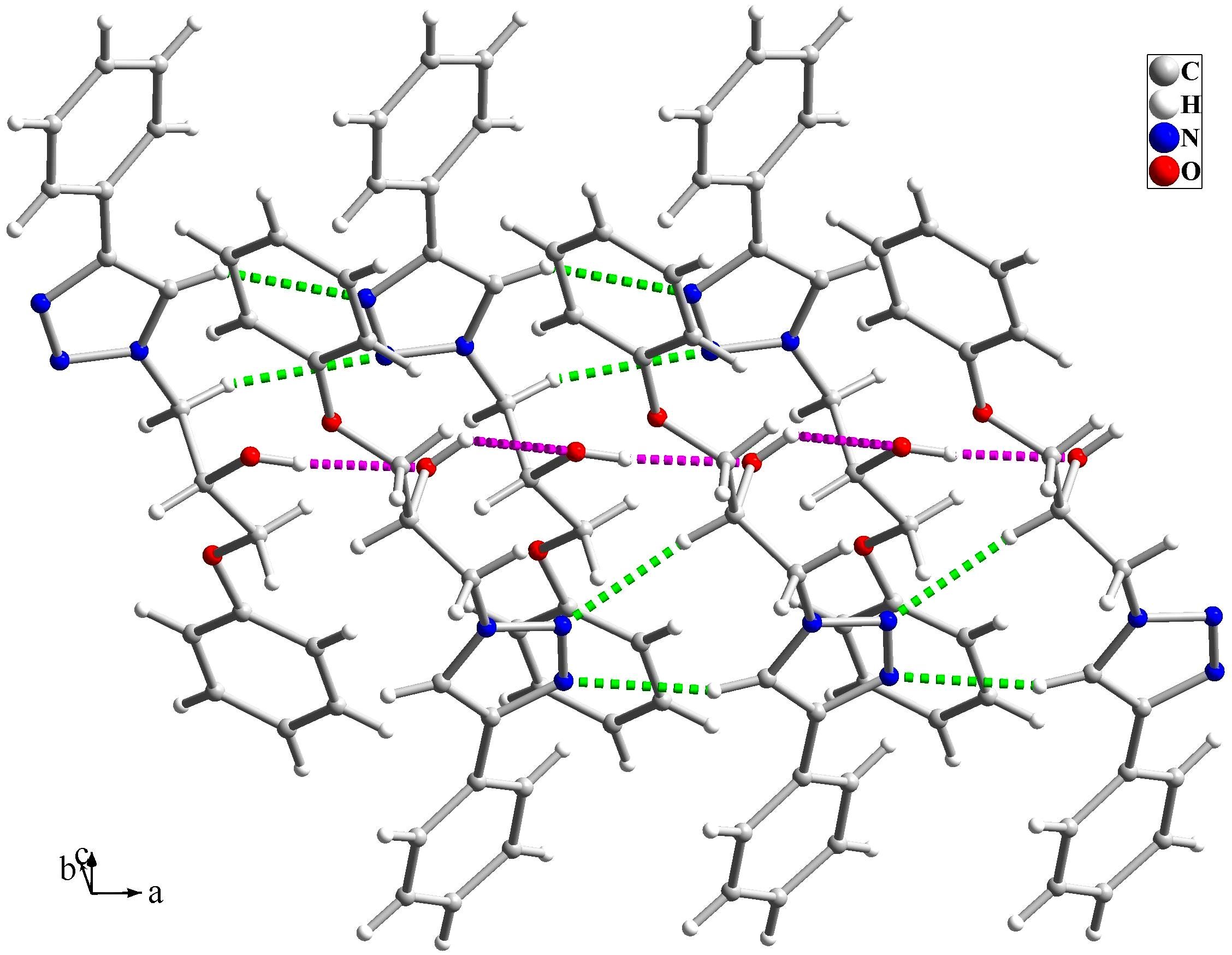


Fig. S10: intermolecular interactions in the crystal structure of **T1**


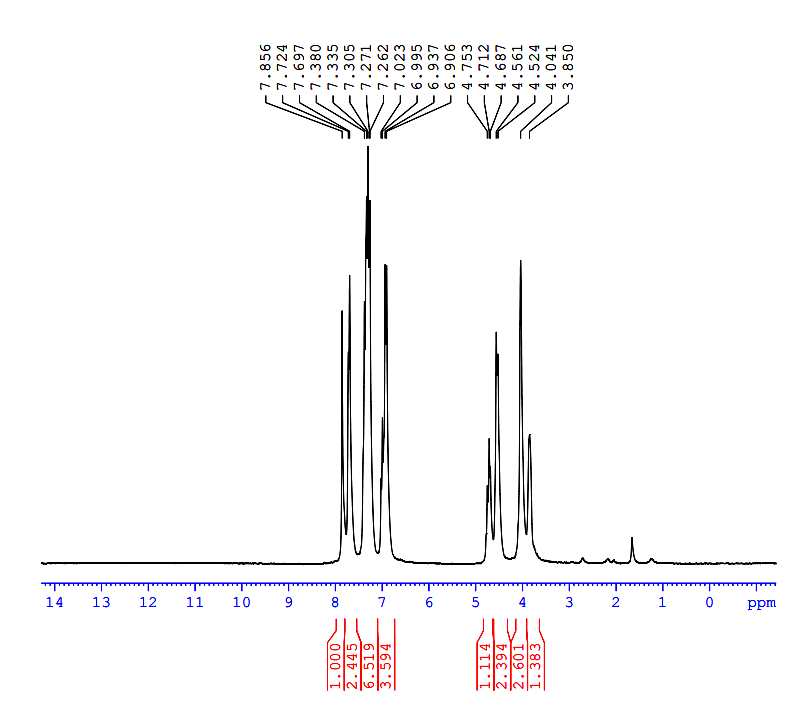


Fig. S11: ^1^ H NMR spectrum of the synthesized **T1** in CDCl_3_ solution


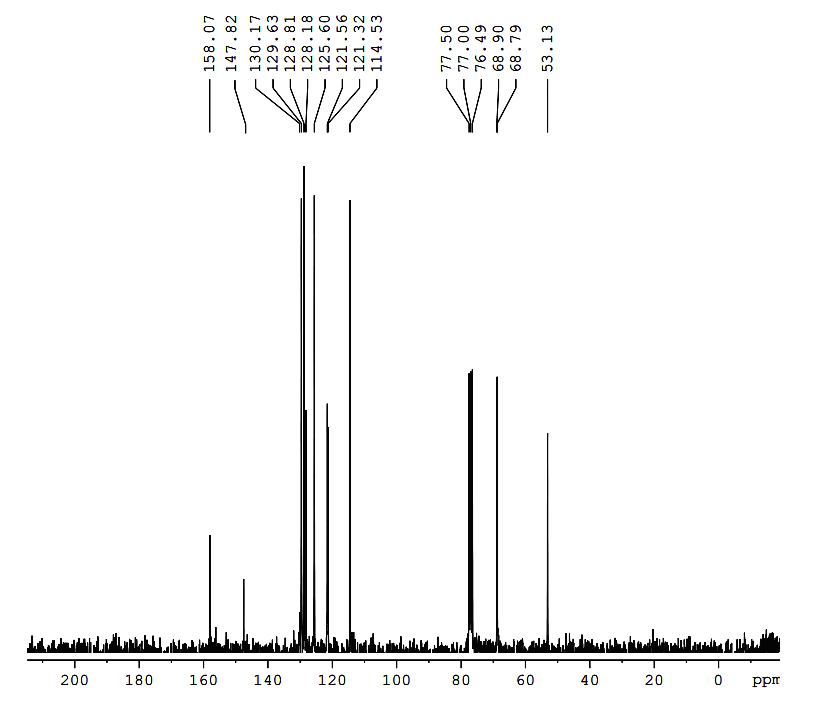


Fig. S12: ^13^ C NMR spectrum of **T1** in CDCl_3_ solution

Table S1: Hydrogen bonds for S-[Cu(HL)Cl_2_] (Å, °).

| **D—H···A** | **D—H** | **H···A** | **D···A** | **D—H···A** |
| --- | --- | --- | --- | --- |
| **O10—H10C···O11^i^** | 0.82 | 1.84 | 2.652(3) | 169 |
| **O11—H11C···Cl1^ii^** | 0.82 | 2.21 | 3.020(2) | 170 |
| **Symmetry codes: (i) −x−1/2, −y, z+1/2; (ii) x−1/2, −y−1/2, −z−2.** | | | | |

Table S2: Hydrogen bonds for R-[Cu(HL)Cl_2_] (Å, °).

| **D—H···A** | **D—H** | **H···A** | **D···A** | **D—H···A** |
| --- | --- | --- | --- | --- |
| **C2—H2A···Cl1^i^** | 0.93 | 2.99 | 3.654(6) | 130 |
| **O10—H10C···O11^ii^** | 0.82 | 1.83 | 2.646(5) | 177 |
| **O11—H11C···Cl1^iii^** | 0.82 | 2.20 | 3.015(4) | 171 |
| **Symmetry codes: (i) −x+1, y+1/2, −z+1/2; (ii) −x+3/2, −y+1, z−1/2; (iii) x+1/2, −y+3/2, −z+1.** | | | | |

Table S3: Hydrogen bonds for S-[Cu(HL)Br_2_] (Å, °).

| **D—H···A** | **D—H** | **H···A** | **D···A** | **D—H···A** |
| --- | --- | --- | --- | --- |
| **C2—H2A···Br1** | 0.95 | 3.03 | 3.568(6) | 118 |
| **C2—H2A···Br2^i^** | 0.95 | 3.03 | 3.738(6) | 132 |
| **C7—H7A···Br1^ii^** | 0.95 | 3.05 | 3.823(6) | 140 |
| **C10—H10A···Br2** | 0.99 | 3.12 | 3.685(6) | 118 |
| **O10—H10C···Br2** | 0.91 | 3.09 | 3.557(5) | 114 |
| **C11—H11B···Br1^iii^** | 0.99 | 3.10 | 3.976(6) | 148 |
| **O11—H11C···Br2^iv^** | 0.84 | 2.34 | 3.177(4) | 173 |
| **Symmetry codes: (i) −x+1, y−1/2, −z+1/2; (ii) x−1/2, −y+1/2, −z+1; (iii) −x+3/2, −y+1, z+1/2; (iv) x+1/2, −y+1/2, −z+1.** | | | | |

Table S4: Selected bond lengths (Å) and angles (°) in the structure of **T1**

| **Bond** | **Length/Å** | **Bond** | **Length/Å** |
| --- | --- | --- | --- |
| C1A—C2A | 1.391(14) | C1B—C2B | 1.383(14) |
| C1A—C6A | 1.392(13) | C1B—C6B | 1.384(13) |
| C2A—C3A | 1.369(15) | C2B—C3B | 1.368(14) |
| C3A—C4A | 1.360(16) | C3B—C4B | 1.366(15) |
| C4A—C5A | 1.392(15) | C4B—C5B | 1.380(14) |
| C5A—C6A | 1.384(13) | C5B—C6B | 1.398(13) |
| C6A—C7A | 1.459(12) | C6B—C7B | 1.460(13) |
| C7A—C11A | 1.365(12) | C7B—C11B | 1.361(13) |
| C7A—N8A | 1.372(11) | C7B—N8B | 1.354(12) |
| N8A—N9A | 1.325(10) | N8B—N9B | 1.346(11) |
| N9A—N10A | 1.350(9) | N9B—N10B | 1.350(11) |
| N10A—C11A | 1.330(11) | N10B—C11B | 1.360(12) |
| N10A—C12A | 1.445(10) | N10B—C12B | 1.439(13) |
| C12A—C13A | 1.523(13) | C12B—C13B | 1.449(13) |
| C13A—O14A | 1.418(11) | C13B—O14B | 1.429(12) |
| C13A—O14C | 1.47(4) | C13B—C15B | 1.554(15) |
| C13A—C15A | 1.512(12) | C15B—O16B | 1.395(12) |
| C15A—O16A | 1.430(10) | O16B—C17B | 1.359(12) |
| O16A—C17A | 1.376(11) | C17B—C18B | 1.373(14) |
| C17A—C22A | 1.374(13) | C17B—C22B | 1.393(13) |
| C17A—C18A | 1.375(13) | C18B—C19B | 1.399(15) |
| C18A—C19A | 1.395(14) | C19B—C20B | 1.352(16) |
| C20A—C21A | 1.368(14) | C20B—C21B | 1.356(15) |
| C19A—C20A | 1.363(15) | C21B—C22B | 1.381(14) |
| **Angle** | **deg/º** | **Angle** | **deg/º** |
| C2A—C1A—C6A | 120.4(11) | C20A—C21A—C22A | 119.4(11) |
| C3A—C2A—C1A | 120.4(12) | C17A—C22A—C21A | 120.6(11) |
| C4A—C3A—C2A | 119.6(13) | C2B—C1B—C6B | 119.6(10) |
| C3A—C4A—C5A | 121.1(13) | C3B—C2B—C1B | 121.8(11) |
| C6A—C5A—C4A | 120.1(12) | C2B—C3B—C4B | 118.7(12) |
| C5A—C6A—C1A | 118.5(10) | C3B—C4B—C5B | 121.2(12) |
| C5A—C6A—C7A | 122.1(10) | C4B—C5B—C6B | 120.0(10) |
| C1A—C6A—C7A | 119.4(10) | C5B—C6B—C1B | 118.7(10) |
| C11A—C7A—N8A | 107.0(9) | C5B—C6B—C7B | 121.2(10) |
| C11A—C7A—C6A | 131.2(9) | C1B—C6B—C7B | 120.1(10) |
| N8A—C7A—C6A | 121.7(9) | C11B—C7B—N8B | 108.9(10) |
| N9A—N8A—C7A | 109.5(8) | C11B—C7B—C6B | 130.9(10) |
| N8A—N9A—N10A | 105.9(7) | N8B—C7B—C6B | 120.2(10) |
| C11A—N10A—N9A | 111.5(8) | N9B—N8B—C7B | 109.4(9) |
| C11A—N10A—C12A | 127.9(8) | N8B—N9B—N10B | 105.5(9) |
| N9A—N10A—C12A | 120.4(8) | N9B—N10B—C11B | 111.5(9) |
| N10A—C11A—C7A | 106.1(9) | N9B—N10B—C12B | 116.6(10) |
| N10A—C12A—C13A | 112.4(8) | C11B—N10B—C12B | 131.5(10) |
| O14A—C13A—O14C | 109.3(19) | N10B—C11B—C7B | 104.8(9) |
| O14A—C13A—C15A | 109.3(8) | N10B—C12B—C13B | 114.6(10) |
| O14C—C13A—C15A | 107.7(18) | O14B—C13B—C12B | 110.5(10) |
| O14A—C13A—C12A | 110.2(9) | O14B—C13B—C15B | 108.5(10) |
| O14C—C13A—C12A | 109(2) | C12B—C13B—C15B | 110.0(10) |
| C15A—C13A—C12A | 111.0(8) | O16B—C15B—C13B | 106.3(10) |
| O16A—C15A—C13A | 106.6(8) | C17B—O16B—C15B | 117.7(9) |
| C17A—O16A—C15A | 118.3(7) | O16B—C17B—C18B | 115.6(10) |
| C22A—C17A—O16A | 115.6(9) | O16B—C17B—C22B | 124.7(10) |
| C22A—C17A—C18A | 120.7(10) | C18B—C17B—C22B | 119.6(11) |
| O16A—C17A—C18A | 123.7(10) | C17B—C18B—C19B | 118.3(12) |
| C17A—C18A—C19A | 117.8(11) | C20B—C19B—C18B | 121.9(12) |
| C20A—C19A—C18A | 121.7(12) | C19B—C20B—C21B | 119.7(12) |
| C21A—C20A—C19A | 119.9(11) | C20B—C21B—C22B | 120.5(12) |

Table S5. Hydrogen bond interactions in the crystal structure of **T1**

| **D—H···A** | **D—H** | **H···A** | **D···A** | **D—H···A** |
| --- | --- | --- | --- | --- |
| C11*A*—H11*A*···N8*A*^i^ | 0.93 | 2.62 | 3.435 (12) | 146 |
| O14*C*—H13*A*···N10*A* | 0.52 | 2.70 | 2.97 (4) | 117 |
| O14*C*—H13*A*···O14*A* | 0.52 | 1.95 | 2.36 (4) | 138 |
| O14*A*—H13*C*···N10*A* | 0.46 | 2.62 | 2.824 (10) | 112 |
| O14*A*—H13*C*···O14*C* | 0.46 | 2.02 | 2.36 (4) | 135 |
| O14*A*—H14*A*···O14*B* | 0.80 | 2.30 | 3.016 (11) | 149 |
| O14*C*—H14*C*···O14*B*^ii^ | 0.82 | 1.79 | 2.61 (4) | 174 |
| C15*A*—H15*A*···O14*C*^i^ | 0.97 | 2.63 | 3.43 (5) | 141 |
| C15*A*—H15*B*···N8*B*^ii^ | 0.97 | 2.61 | 3.487 (13) | 150 |
| C11*B*—H11*B*···N8*B*^ii^ | 0.93 | 2.56 | 3.454 (13) | 163 |
| C13*B*—H13*B*···N9*B*^ii^ | 0.98 | 2.51 | 3.471 (15) | 167 |
| O14*B*—H14*B*···O14*A* | 0.84 | 2.50 | 3.016 (11) | 121 |
| **Symmetry codes: (i) −x+1, y−1/2, −z+1/2; (ii) x−1/2, −y+1/2, −z+1; (iii) −x+3/2, −y+1, z+1/2; (iv) x+1/2, −y+1/2, −z+1.** | | | | |

1. CrysAlisPro, Oxford Diffraction/Agilent Technologies UK Ltd, Yarnton, England. [↑](#endnote-ref-2)
2. G.M. Sheldrick, Crystal structure refinement with SHELXL. Acta Cryst. 2015, C71, 3-8. [↑](#endnote-ref-3)
